# An in-depth comparison of linear and non-linear joint embedding methods for bulk and single-cell multi-omics

**DOI:** 10.1093/bib/bbad416

**Published:** 2023-11-28

**Authors:** Stavros Makrodimitris, Bram Pronk, Tamim Abdelaal, Marcel Reinders

**Affiliations:** Delft Bioinformatics Lab, Delft University of Technology, Street, Postcode, State, Country; Department of Medical Oncology, Erasmus University Medical Center, Street, Postcode, State, Country; Department of Clinical Genetics, Erasmus University Medical Center, Street, Postcode, State, Country; Delft Bioinformatics Lab, Delft University of Technology, Street, Postcode, State, Country; Delft Bioinformatics Lab, Delft University of Technology, Street, Postcode, State, Country; Department of Radiology, Leiden University Medical Center, Street, Postcode, State, Country; Leiden Computational Biology Center, Leiden University Medical Center, Street, Postcode, State, Country; Delft Bioinformatics Lab, Delft University of Technology, Street, Postcode, State, Country; Leiden Computational Biology Center, Leiden University Medical Center, Street, Postcode, State, Country

**Keywords:** multi-omics, joint embedding, dimensionality reduction, neural networks

## Abstract

Multi-omic analyses are necessary to understand the complex biological processes taking place at the tissue and cell level, but also to make reliable predictions about, for example, disease outcome. Several linear methods exist that create a joint embedding using paired information per sample, but recently there has been a rise in the popularity of neural architectures that embed paired -omics into the same non-linear manifold. This work describes a head-to-head comparison of linear and non-linear joint embedding methods using both bulk and single-cell multi-modal datasets. We found that non-linear methods have a clear advantage with respect to linear ones for missing modality imputation. Performance comparisons in the downstream tasks of survival analysis for bulk tumor data and cell type classification for single-cell data lead to the following insights: First, concatenating the principal components of each modality is a competitive baseline and hard to beat if all modalities are available at test time. However, if we only have one modality available at test time, training a predictive model on the joint space of that modality can lead to performance improvements with respect to just using the unimodal principal components. Second, -omic profiles imputed by neural joint embedding methods are realistic enough to be used by a classifier trained on real data with limited performance drops. Taken together, our comparisons give hints to which joint embedding to use for which downstream task. Overall, product-of-experts performed well in most tasks and was reasonably fast, while early integration (concatenation) of modalities did quite poorly.

## INTRODUCTION

In the past years, there has been a tendency to produce more and more multi-modal -omics data [1], i.e. to measure multiple different data modalities from the same sample. This has enabled the discovery of complex biological mechanisms and lead to deeper understanding of biological processes (e.g. [2, 3]). For example, with joint profiling of genetic and transcriptomic data from the same individuals we can uncover eQTLs (expression quantitative trait loci), i.e. genetic variants that influence the expression of particular genes [4].

A prime example of such multi-modal datasets is that of The Cancer Genome Atlas (TCGA), which contains mutation, gene expression, DNA methylation and copy number profiles from the same tumor sample for thousands of primary tumors from different cancer types [5]. More recent advances in single-cell technology have enabled the simultaneous profiling of different -omics from the same single cell, for example gene expression and DNA methylation [6] or gene expression and protein expression [7]. A review of multi-modal single-cell technologies can be found in [8].

This rapid progress in our ability to generate data has naturally increased the need for computational tools to deal with the challenges of analyzing increasing amounts of multi-modal data. One popular class of such tools is called joint dimensionality reduction or joint embedding and involves projecting all modalities into the same lower-dimensional space [9–11]. The ’joint space’ then attempts to encode the information shared by all modalities and filter out modality-specific signals. This not only reduces the effects of experimental noise, but also helps us uncover relationships between modalities.

Several joint embedding methods have been proposed and many of them have been especially designed for -omics data. The majority of such methods are extensions of single modality dimensionality reduction such as factor analysis or principal components analysis. For example, Multi-Omics Factor Analysis (MOFA+) [10] is a generalization of probabilistic Principal Components Analysis (PCA) for multi-modal data, as it uses variational inference to find a linear projection that minimizes the total reconstruction error of all modalities. Through the use of appropriate prior distributions, MOFA+ also tries to learn a ’sparse’ space with a small number of factors and a small number of features contributing to each factor. Other methods do not focus on reconstruction, but to find projections that maximize a criterion. For instance, Multiple Co-Inertia Analysis (MCIA) [11] maximizes the covariance between the input profiles and the latent representation, while AJIVE [12] finds the directions of maximum variance for the concatenated principal components of all modalities.

Recently, the use of neural networks and deep learning for dimensionality reduction has gained popularity in several domains, including computational biology and mainly single-cell transcriptomics [13]. This is motivated by the fact that neural networks can identify non-linear patterns in the data which are expected to be present in -omics data (e.g. synthetic lethality [14] or enhancer synergy [15]). A widely-used model in such settings is the Variational AutoEncoder (VAE) [16], which uses a neural network to learn a probabilistic mapping from an input profile to a latent variable (encoder), while a second network (decoder) learns the inverse mapping (from the latent variables to the input). VAEs have also been used in multi-modal settings. For example, totalVI learns a joint embedding of gene and protein expression profiles from Cellular Indexing of Transcriptomes and Epitopes (CITE)-Seq data by concatenating the two feature sets (early integration) [17]. Another interesting example is UniPort, which learns an embedding of only one (pre-defined) modality, but learns a joint latent space by requiring that both modalities can be reconstructed from these latent features [18].

Advances in multi-modal deep learning from other fields, such as computer vision, can also be applied in computational biology. One such model which also uses VAEs is called Product of Experts (PoE) autoencoder [19]. It uses one encoder for each input modality and combines the different encoder outputs to yield the final joint embedding. The formulation of PoE makes it usable in settings where some modalities are not observed. The Mixture of Experts (MoE) [20] autoencoder modified the way the single-modality embeddings are combined for the joint embedding by (a) using a mixture instead of multiplying the single-modality posteriors, (b) changing the latent distribution from normal to Laplace and (c) employing a gradient estimator with lower variance [20]. These lead to improved performance with respect to PoE [20], although the superiority of MoE is not that well-established in the machine learning literature [21]. Both models have been applied in single-cell multi-omic datasets (e.g. [22, 23]). For example, Minoura *et al*. [23] used MoE to identify regulatory relationships between gene expression and chromatin accessibility, as well as to predict one modality from the other.

The large number of available methods calls for comparisons between them. Cantini *et al*. compared nine *linear* joint embedding methods at various tasks using both bulk and single-cell data [9]. The main finding of this study was that there was large variability on the ranking of methods depending on the task, but a few methods, such as MCIA [11] tended to rank near the top for most tasks. Another observation made by the authors was that most of the tested methods were designed for bulk data, but nevertheless also performed reasonably well on single-cell data. Next to being restricted to linear methods, a limitation of this study is that the authors used a single-cell dataset of only 206 cells, which is too small to be representative of modern single-cell experiments, which enable multi-omic single-cell profiling at much larger scales. For example, with SNARE-Seq and ISSAAC-seq it is possible to measure gene expression and chromatin accessibility in over ten thousand cells [24, 25], and a recent CITE-Seq study profiled gene and protein expression in more than half a million cells in a single experiment [26]. Therefore, possible differences in scalability among these methods were not taken into account. In another study, Brombacher *et al*. compared the performance of several deep-learning-based joint representation learners as a function of the number of cells in the datasets [27]. This study was focused on single-cell data and did not compare to any well-established linear methods.

**Figure 1:**
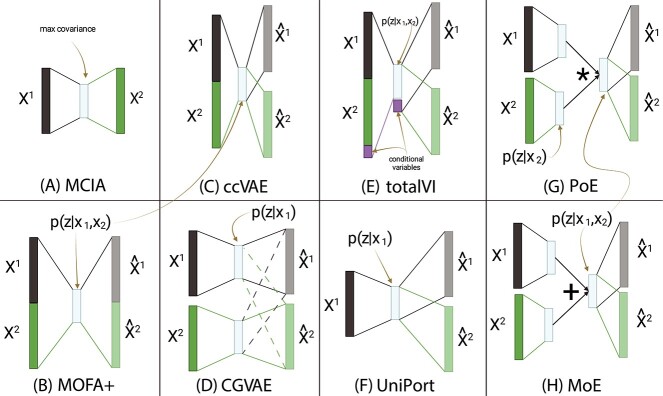
Schematic representation of the eight joint representation learning models benchmarked in this work. On the left, the linear methods: MCIA (**A**) and MOFA+ (**B**); in the middle the baseline non-linear methods: ccVAE (**C**) and CGVAE (**D**); and on the right the existing non-linear methods: totalVI (**E**), UniPort (**F**), PoE (**G**) and MoE (**H**). Yellow arrows indicate important features of each method such as the latent embeddings conditioned on one or both modalities.

**Figure 2:**
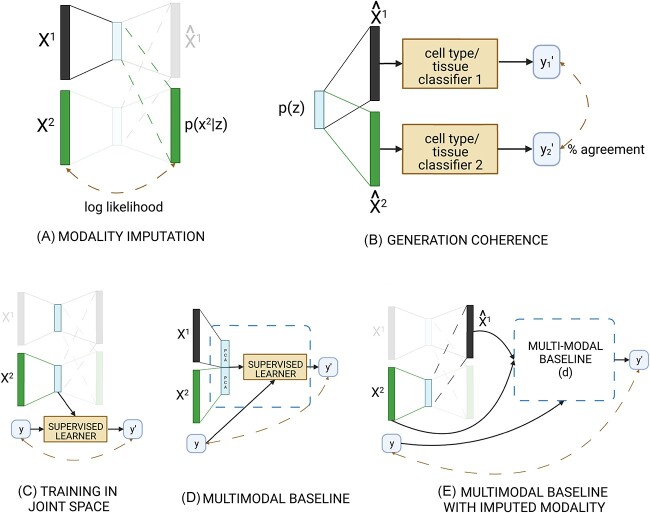
Sketch of our evaluation schemes for (**A**) modality imputation, (**B**) generation coherence, (**C–E**) downstream supervized tasks. Panel (C) shows the training of a classifier in the joint space of one modality, and (D) the baseline method. In panel (E), we impute the missing modality and feed the measured and the imputed profile into the baseline of panel (D).

In this paper, we compare several neural network architectures for joint representation learning to each other as well as to two popular linear methods that also showed promise according to Cantini *et al*. [9]. An overview of the methods included in the comparison is shown in Figure 1 and Table S1. We evaluate the models’ ability to impute missing modalities, to learn a coherent latent space, and to perform well on downstream tasks using both bulk and single-cell data. Moreover, when appropriate, we employ simple baselines that do not make any use of joint dimensionality reduction to put the observed performances into perspective (Figure 2). We show that these baselines are sometimes tough to beat, especially for downstream supervized tasks. Additionally, we show that non-linear methods are needed for accurate imputation of missing modalities and that these methods generated -omic profiles are realistic enough to be used by classifiers expecting real profiles.

## RESULTS

### Non-linear methods show superior imputation on TCGA

We first compared two linear joint embedding methods, MCIA ([11], Figure 1A) and MOFA+ ([10], Figure 1B) against five non-linear, neural-network-based joint dimensionality reduction methods using the TCGA data. We included two simple non-linear architectures as baseline: concatenated VAE (ccVAE, Figure 1C) works by concatenating the modalities and feeding them to a common encoder. When imputing one modality from the other, all inputs of the missing modality are set to zero as in totalVI [17] (Figure 1E). Cross-Generating VAE (CGVAE, Figure 1D) uses separate encoders and learns a joint space by (a) forcing each encoder’s output to be able to reconstruct all modalities, and (b) an additional loss term penalizing the difference between the learnt embeddings of each modality based on Wasserstein distance (Materials and Methods). We also benchmarked three existing VAE-based joint embedding methods, namely UniPort [18] (Figure 1F), product of experts (PoE, [19], Figure 1G) and mixture of experts (MoE, [20], Figure 1H). MCIA and MOFA+ cannot be readily applied to new unseen samples (out-of-sample extension), but since they are linear methods, we enabled this by fitting linear mappings from the input to the embedding space and vice-versa. See Materials and Methods for more details about all methods and their training.

**Figure 3:**
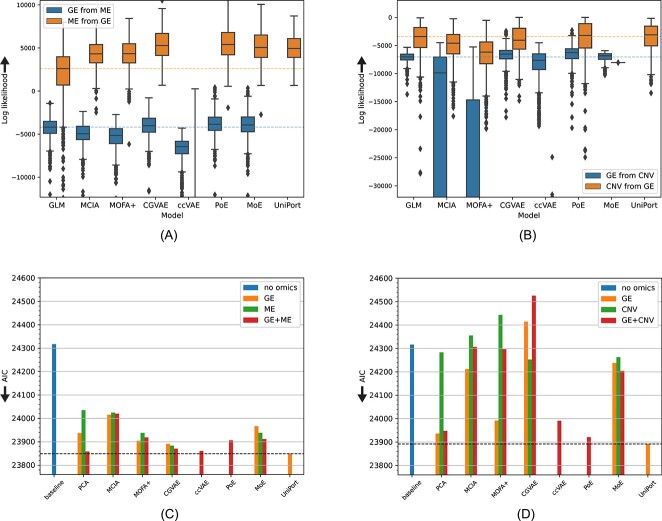
Missing modality imputation and survival analysis performance on the TCGA dataset. (**A**) Imputing GE from ME and vice-versa, and (**B**) GE from CNV and vice-versa. Performance is measured in both cases as the log-likelihood ($y-$axis, equation 1) of the 844 test samples given the predictions of each model ($x-$axis) for those data (higher is better, signified by upwards-pointing arrows). The dashed horizontal lines represent the performance of the GLM baseline. The median log-likelihood for ccVAE at predicting ME from GE is –70 987.14 and CNV from GE –74 111.33 (not shown to ease visualization of the remaining models). Test samples whose log-likelihood is further than 1.5 times the interquartile range from the median sample are marked as outliers. (**C**) Predicting progression-free survival using the joint embeddings on the TCGA dataset with GE+ME. The $y-$axis shows the AIC (lower is better as signified by downwards arrow) achieved by different models trained in the joint space of GE and ME using either one (GE, ME) or two modalities. The performance of baseline models using PCA as well as only the covariates is also shown, while the best performance achieved by any method is indicated by a dashed line. The $y-$axis is scaled to include all observed AIC values. (**D**) As in (c), but for the GE+CNV dataset.

Using 6752 samples from 33 tumor types from the TCGA with gene expression (GE), methylation (ME) and Copy Number Variation (CNV) data available, we learned joint embeddings for GE + ME and GE + CNV, and evaluated the ability to impute a missing modality using a held-out test set of 844 samples (Figure 2A). We also compared against a baseline Generalized Linear Model (GLM) trained to perform regression from one modality to the other without any joint embedding. The results are summarized in Figure 3A and B and Tables S2 and S3, where each model’s performance is measured as the log-likelihood of the test data given the model’s predictions (Equation 1, Materials and Methods). UniPort only encodes one modality (reference) (Figure 1F) and learns a joint space that can be used to reconstruct both modalities using separate decoders. We used GE as the reference modality here, which means that with UniPort we could only impute ME or CNV from GE, but not vice versa.

Despite the fact that we also tested hyperparameter configurations without any hidden layers (using a validation set of 844 samples, independent of the training and test sets), we found that, for all five models, the optimal configuration for this task, based on the validation set, was non-linear with either one (ccVAE, UniPort, PoE) or two (CGVAE, MoE) hidden layers. The selection of the remaining hyperparameters was highly dependent on the choice of model and dataset (Table S4).

Figure 3A and B shows that both MCIA and MOFA+ failed to outperform the simple regression baseline in three out of the four cases and only performed well at imputing DNA methylation patterns from gene expression. Furthermore, concatenating a measured modality with a vector of zeros’s for the missing modality and passing the data through ccVAE for imputation lead to very bad performances, considerably worse than all other methods (Figure 3A and B).

CGVAE, PoE, MoE and UniPort performed better than the other three methods, but only PoE was significantly better than the GLM baseline in all four cases (FWER < 0.05, Wilcoxon test). CGVAE and MoE offered significant improvements with respect to the baseline in general, but they both failed at predicting CNVs from GE (Figure 3B). UniPort was also significantly better than the baseline at predicting ME and CNV from GE (FWER < 0.05, Wilcoxon test).

Overall, we observed large improvements in imputation performance with respect to the baseline for the prediction of methylation from gene expression (almost 2-fold increase in log-likelihood), while in most of the remaining cases the differences were marginal (albeit statistically significant). Taken together, we can conclude that PoE is the best performing method in the imputation task.

We additionally compared the coherence of the latent space of the different joint embedding methods by testing whether decodings of the same point in the latent space are classified as the same cancer type by a neural network trained to predict the cancer type using the measured training data (Figure 2B). The predictive performance of the CNV-based cancer type classifier was quite bad with respect to those of the GE- and ME-based classifiers, therefore we restricted the comparison only to the GE and ME dataset (Table S5). Remarkably, PoE and MoE (the top-performing methods at imputation) had the two worst latent space coherence performances (Table 1). The latent space of ccVAE was more coherent, giving decodings from the same cancer type in 75% of the cases, second to CGVAE with 81%. UniPort and MOFA+ ranked third and fourth respectively, performing slightly better than PoE (Table 1). Note that MCIA is not a generative model, so we cannot sample from its latent space and therefore it is not included in this experiment.

**Table 1:** Generation coherence evaluation. Coherence is measured as the fraction of times the decodings of random points from the latent space are from the same class (cancer type for TCGA, cell type for RNA+ATAC-Seq and CITE-Seq). Higher is better. Note that MCIA is not generative and cannot be evaluated in this setting

Model	TCGA (GE+ME)	RNA+ATAC	CITE-seq
MCIA	N/A	N/A	N/A
MOFA+	0.53	0.84	0.62
CGVAE	0.81	0.85	0.56
ccVAE	0.75	0.89	0.49
TotalVI	N/A	N/A	0.47
UniPort	0.57	0.83	0.33
PoE	0.50	0.74	0.30
MoE	0.27	0.78	0.50

From these results, we conclude that there is a discrepancy between imputation and coherence as no architecture is very good at both at the same time. On the other hand, we found that in all tasks the top-performing method was a non-linear one.

### Limited practical utility of joint embedding methods for survival analysis

We then tested whether the pretraining of joint dimensionality reduction would lead to performance gains in the supervized downstream task of survival analysis (Figure 2C and D). Specifically, we used the latent features learned by each model to fit a pan-cancer Cox proportional hazards model to predict progression-free survival using age, gender and cancer type as covariates (Materials and Methods). We tested these methods with either one modality as input (and then take the latent representation related to that modality), as well as with two modalities as input.

As baseline, we used a model with only the covariates mentioned above. Additionally, we compared to three more simple models: two single-omic models based on the 32 principal components of each data modality (PCA, orange and green bars in Figure 3C and D) and a multi-omic model whose input was the concatenation of the 32 principal components of the two modalities (Figure 2D, red PCA bars in Figure 3C and D).

We evaluated using the Akaike Information Criterion (AIC). The AIC measures the quality of a model’s fit to the data by taking the number of model parameters into account, as it is in principle easier for models with more parameters to overfit to the training data. The results of this experiment (the lower the AIC the better) are shown in Figure 3C and D and Tables S6–S7.

Our baseline model using only the patients’ sex, age and cancer type (and no -omics) information already provided a statistically significant fit (FWER < 0.05) compared to a null model with only an intercept (result not shown). The inclusion of the 32 principal components of gene expression or methylation (PCA) lead to large improvements (Figure 3C). In fact, combining the principal components of these modalities outperformed all but one joint embedding method. PCA on the copy number data only yielded a minor reduction in AIC with respect to the baseline (Figure 3D). Gene expression is the most predictive single modality of the three in this task.

Cox models trained on the latent space of joint embedding methods using both gene expression and methylation (GE+ME) did improve upon the baseline, but were all outperformed by PCA, with ccVAE, CGVAE and PoE ranking second, third and fourth respectively when both modalities are included (Figure 3C). MCIA had the worst performance in this setting, but still provided a significant fit (FWER < 0.05).

As for models trained on the latent space of gene expression and copy number (GE+CNV, Figure 3D), joint pre-training sometimes proved detrimental as CGVAE provided a very poor fit, comparable to what could be expected by chance (FWER > 0.05) and MOFA+ and MCIA barely outperformed the covariate baseline. However, PoE did outperform all other methods, with PCA second and ccVAE ranking third (Figure 3D).

When we restrict our comparison to methods that only use one modality, we hypothesized that a joint embedding will be beneficial, as the joint pre-training can provide additional information from other modalities. For gene expression (the most informative single modality), this was in general not the case, as only three of the seven joint embedding methods (MOFA+, CGVAE and UniPort) trained on the joint space of gene expression and methylation (GE+ME) outperformed training only on GE data. UniPort’s gene expression embeddings gave the best predictions, even outperforming all methods that use both GE and ME. Cox models that use the gene expression data embedded in the GE+CNV joint space performed worse than the Cox model trained on the PCA of the GE data, with the exception of UniPort, which was again the top-performing method. This implies that the common information between gene expression and copy number is at large not related to disease progression, possibly because CNV data have relatively small prognostic power (Figure 3D). Nevertheless, UniPort did manage to capture information related to survival from this dataset and is potentially a promising method for jointly embedding bulk data along with PoE.

On the other hand, training a model on the joint methylation space (GE+ME) does improve the AIC performance when comparing to training on only ME data for all joint models (Figure 3C). Moreover, gene expression informed by methylation outperformed gene expression informed by copy number for all five models (Figure S1). These results indicate that the joint embedding space of gene expression and methylation does encode information about the metastatic potential of tumors.

Finally, we devised a method to test whether each latent factor encodes for joint signal shared by both modalities or is modality-specific (Quantification of Joint Signal, Supplementary material). This method works by estimating the mutual information between a latent factor and each input data modality and is applicable only to VAE-like methods that can encode each modality separately. Applying it on the models we trained on the TCGA data, we found that in many of the cases, the latent space is a mixture of joint and modality-specific factors, although we did observe models with mostly joint factors, with factors of PoE being slightly more associated with both modalities than other methods (Figure S2A and B). Our analysis also showed that most latent factors of the MoE model trained on the GE+CNV dataset were not significantly associated with either modality, which might be an explanation of the relatively poor performance of MoE on this dataset.

### Similar patterns hold for paired single-cell RNA and ATAC-Seq data

To evaluate the methods on single-cell data, we first used a dataset of 10 412 peripheral blood mononuclear cells (PBMCs) containing both gene expression and chromatin accessibility profiles for each cell. The dataset was split into training (80%), validation (10%) and test sets (10%) stratified per cell type.

We found that PoE, MoE and UniPort did the best at imputation, outperforming the baseline (Figure S3A, Table S8), while ccVAE and CGVAE had the best generation coherence, although all other methods followed closely (Table 1). In all models, non-linear architectures with two hidden layers were selected based on the validation loss and only the PoE model used dropout and batch normalization (Table S9).

We also compared the pre-trained models on the supervized task of cell type classification (Figure 2C), using the same train-validation-test split as above. For each model, we trained two cell type classifiers: a linear SVM and a two-layer MLP (Materials and Methods). The input features for the classifiers were the cell embeddings of either RNA ($q(z|x_{1})$), ATAC ($q(z|x_{2})$), or both ($q(z|x_{1},x_{2})$). This specific 20-class problem is rather simple, as a MLP trained on the 32 PCs of the RNA data achieved a Matthews Correlation Coefficient (MCC) of 0.90, while concatenating the 32 PCs of both modalities was even better with a MCC of 0.93 (Figure S3B and C, Table S10). None of the tested joint embedding methods was able to do better than these numbers; the best MCC values were achieved by MOFA+ (0.87) and PoE (0.86) using RNA-only embeddings.

In summary, for this single-cell dataset, Uniport and PoE performed well at imputation, while ccVAE did quite poorly and PCA was a very strong baseline for downstream supervized classification. These results in all three tasks (imputation, coherence, classification) are very much in line with our findings on the bulk TCGA data. What was different, is that most of the latent factors learned by the models in this dataset were not found to share significant information with both modalities according to our mutual information-based test. Instead, they were mostly modality-specific (Figure S2C).

### Neural architectures impute and scale better on a large CITE-Seq dataset

We then compared the same seven joint embedding methods plus totalVI on a CITE-Seq dataset profiling PBMCs from eight different individuals [7]. We used six individuals for training (117 730 cells), one for validation (16 718 cells), and one for testing (17 646 cells). The dataset also contains cell type annotations for each cell. These annotations are provided at three different levels of granularity: level-1 is the most coarse labeling with eight different classes, level-2 uses 30 classes, and level-3 is the most fine-grained labeling with 57 classes [7].

The best configurations for the non-linear models were as follows (Table S11): the PoE architecture was composed of two layers, while all others had three. Only totalVI used dropout and totalVI, ccVAE and CGVAE used batch normalization. We tried to train MCIA on this training set with 750 GB of RAM but it did not terminate due to insufficient memory after several computation hours so it is not included in this comparison.

**Figure 4:**
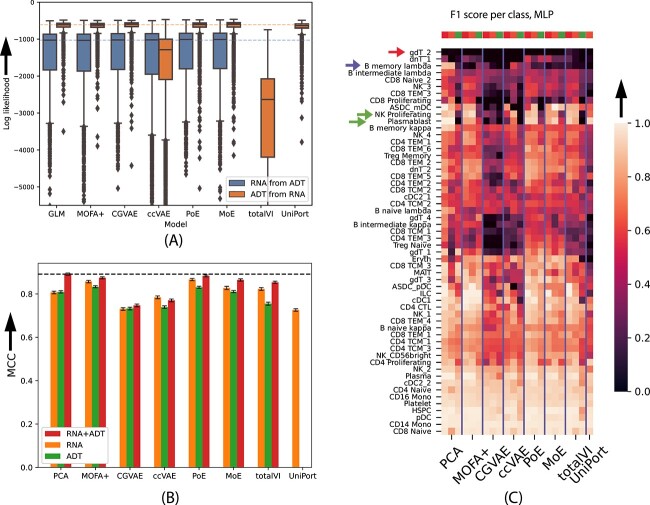
Evaluation on the CITE-Seq dataset. (**A**) Missing modality imputation performance for gene expression (RNA) from protein expression (ADT) and vice-versa. Performance is measured as the log-likelihood ($y-$axis, equation 1) of the test samples (cells) given the predictions of each model ($x-$axis) for those data (higher is better). The distribution of the per-cell log-likelihoods is shown. The dashed horizontal lines represent the performance of the baseline GLM. Cells further than 1.5 times the interquartile range from the median are marked as outliers. (**B**) Cell type classification performance (MCC, $y-$axis, higher is better) achieved by training a multilayer perceptron (MLP) in the joint space of the different models when using: only gene expression (RNA), only protein expression (ADT), and both RNA and ADT data. The error bars denote 95% confidence intervals calculated by bootstrapping the test cells 100 times. (**C**) Per-class (cell type) performance of the same classifiers as in (B). Brighter colors denote a higher per-class F1 score and therefore better performance. For each model we show three columns (RNA+ADT, RNA only, and ADT only, signified by top row, wherever applicable). Arrows show the cell types highlighted in the results. Note that class CD4+ Tem_4 is not present in the test data and therefore not shown in the per-class evaluations (because its precision and recall is always 0 and the F1 score is thus undefined), but it was taken into account when calculating the MCC in (B).

We first compared the methods on missing modality imputation, using a similar GLM-based baseline method as before (Figure 2A). All methods, including the baseline, performed similarly at imputing RNA (RNA) from protein expression (ADT), with MoE having a slight edge over the rest, followed by CGVAE (Figure 4A, Table S12). Pairwise Wilcoxon rank sum tests showed that the performance differences were statistically significant (FWER < 0.05) despite the small absolute differences in median. By inspection of the imputation performance per (level-2) cell type and clustering the cell types based on their median imputation log-likelihood, we found three distinct clusters of cell types (Figure S4). This shows that there is one group of cell types for which all models can perform good imputations. These include common cell types, such as T cells and B cells. For all six methods, there was a positive Spearman correlation between the abundance of a cell type and the accuracy of imputation ($\rho$ = 0.50-0.56, Figure S6). The performances of the six methods across 30 (level-2) cell types were highly correlated, with the pairwise Spearman correlations ranging between 0.959 and 0.997, with a median of 0.966 (Figure S6).

Imputation of ADT from RNA followed the pattern of the TCGA experiments, with MOFA+, ccVAE and totalVI performing worse than the GLM baseline, as did PoE. CGVAE was the top method (Figure 4 and Table S12). In this experiment, we observed much smaller variance in the imputation log-likelihood compared to the RNA for most models (Figure 4A and Figure S5), with most of the outlier cells (i.e. cells for which the imputation is less accurate than the rest) being erythrocytes and platelets. The effect of cell type abundance on the performance was less prominent for imputing ADT, with Spearman correlations ranging from 0.08 to 0.43 for the joint embedding methods, although the GLM was slightly more affected ($\rho$ = 0.48, Figure S6)). Again, we observed high concordance on the performance of the eight methods that imputed ADT from RNA across the 30 cell types ($\rho \in [0.798, 0.987]$, with a median of 0.938, Figure S6)).

It is possible that the observed association between RNA imputation performance and cell type abundance is due to bias towards abundant cell types introduced by the pre-selection of genes, as this association was less prominent for imputing protein expression. To test this hypothesis, we replaced the 5000 most variable genes with 819 (level-2) cell type marker genes, derived using COSG [28] (Supplementary Methods). We found that our set of 5000 most variable genes was significantly enriched for these COSG-derived marker genes, with 694 (85%) overlapping markers (*P*-value < 1e-6, chi-squared test, Table S13). Experiments using the joint embedding models that predict RNA from ADT re-trained on this set of COSG-derived marker genes show consistent improvement (with respect to training on the 5000 highly variable genes) in median imputation performance across all cell types and more so for the less abundant cell types (Figure S7).

To evaluate generation coherence (Figure 2B), we built neural networks that predict the level-2 cell type from the RNA or the ADT data (Table S5). We found that MOFA+ and CGVAE had the highest performance followed by MoE, ccVAE and totalVI. PoE again performed badly in this task (Table 1).

### Multi-modal pre-training partly compensates for an unmeasured modality at test time

For cell type classification (Figure 2C), we used the level-3 labels containing in total 57 different classes (cell types). We used the same data split as for the imputation and followed the experiment set-up of the RNA+ATAC-Seq dataset, comparing two cell type classifiers: a linear SVM and a two-layer MLP (Materials and Methods). As in the previous analyses, we also used another simple method that projects each modality to its 32 principal components and trained the same classifiers (Figure 2D).

We again found that joint unsupervized pre-training on the same dataset does not yield any considerable advantage to downstream performance if both modalities are available at test time, as concatenating the PCs of gene and protein expression (RNA+ADT) gave competitive performance using both classifiers (Figure 4B, Figure S8, Table S14). PoE embeddings did perform slightly better than the PCA baseline using the SVM classifier (Table S14, Figure S8), while CGVAE, ccVAE and UniPort did not perform well in this task. We also observed that – similar to the RNA+ATAC-Seq dataset – the MLP (Figure 4B and C) outperformed the SVM classifier (Figure S8) regardless of the input data, which shows that at least some of the classes are not linearly separable and require non-linear modeling. We thus focus on the MLP classifier onwards.

Next, we evaluated all methods when only one modality is available at test time and compared the models that are pre-trained in the joint space against a classifier trained on the PCs of the single modality. In this setting, there was a drop in the performances with respect to the case when both modalities are always available. However, we observed considerable improvements caused by the joint pre-trainining using MOFA+, PoE, totalVI and MoE, as demonstrated in Figure 4B (orange and green bars). A MLP trained on the RNA latent space of PoE or MOFA+ performed only slightly worse than the best performance that was achieved when having two modalities available. When measuring only protein expression, we obtained similar results, with joint pre-training using MOFA+, PoE, and MoE providing a significant performance gain with respect to the baseline (Figure 4B). Furthermore, ADT data embedded on the joint RNA+ADT space gave worse performance than RNA data embedded on this space for all methods, except for CGVAE, for which the two modalities performed almost equally. This implies that the two joint spaces are not equivalent and that RNA is ’the dominant modality,’ although we corrected for the fact that RNA has more features than ADT (Materials and Methods).

As for the RNA+ATAC-Seq data, most latent factors of CGVAE and ccVAE had significantly large mutual information with only RNA (Figure S2D), while CGVAE and MoE learned one joint factor. PoE, the best-performing of the non-linear models, learned slightly more joint factors (4), 10 of its factors were only sigificantly associated with RNA and another set of 10 factors only with ADT. Finally, MoE and PoE embeddings of ADT data outperformed all other non-linear methods at cell type classification in the absence of RNA information (Figure 4) and this is inline with our observation that these two models had the most factors significantly associated with the ADT data (Figure S2D). These suggest that our mutual information-based test can provide insights into the performance of VAE-based joint embedding methods. Imputation of RNA from ADT on the test set of the CITE-Seq dataset by 6 models trained using 5000 most variable genes (x-axis) of 819 marker genes (y-axis) as RNA features. Performance is measured as the mean log-likelihood of a test cell across all genes. Cells of the same cell type are then aggregated using their median value to reach one average performance for each cell type (dot). The cell type abundance in the dataset is signified by the size of the dot. To gain a better understanding of the performance differences between the different models, we examined the performance per class as measured by the F1 score (Figure 4C). First, we observed that a very rare class of $\gamma \delta$ T cells (gdT cell 2) is very hard to differentiate among other T cell types. Only the PCA of protein expression data and CGVAE with both modalities was able to do better than random on this class (red arrow in Figure 4C). $\lambda$ memory B cells are predicted accurately using PCA on the RNA data, but the performance of the ADT principal components and all joint embedding methods is worse (blue arrow in Figure 4C). This could imply that the information needed to distinguish this cell type is only in the RNA and therefore it is not present in the joint space (RNA+ADT), so joint pre-training is detrimental for the performance for this specific class. On the other hand, proliferating natural killer cells and plasmablasts (green arrows) are also only predicted well by RNA, but embedding RNA on the joint RNA+ADT space of PoE and MoE did not hurt the performance for those classes.

Finally, to test the potential bias towards the set of highly variable genes, we investigated the effect of using the COSG-derived marker genes (Supplementary Methods) on cell type classification. Table S15 contrasts the MCC achieved by the MLP trained in the latent spaces of models trained with the 5000 most variable genes and with the 819 COSG-derived marker genes as RNA features. The use of COSG-derived marker genes lead to improvements in cell type classification for CGVAE, ccVAE and MoE, but not for the two best-performing methods (PoE and PCA baseline).

### Imputed modalities are useful for cell type classification

**Figure 5:**
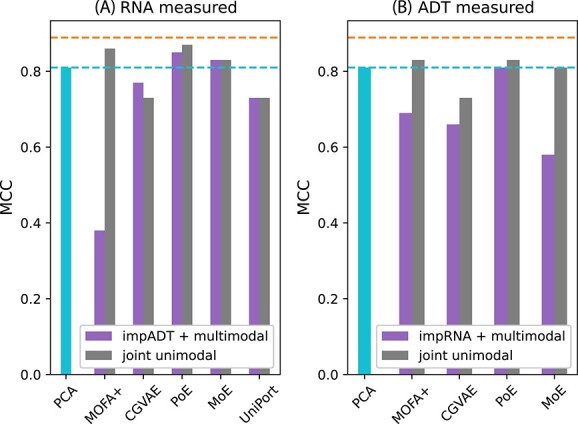
Comparison of training a cell type classifier in the joint space (joint unimodal, Figure 2C) versus using the joint space to impute a missing modality and using a classifier trained on both modalities (impRNA/ADT + multimodal, Figure 2E). Panel (**A**) shows the case when only RNA is available at test time, and (**B**) when only ADT measurements are available at test time. Performance is quantified by the MCC (higher is better). The left-most bar (light blue) and attached dashed line correspond to the performance of a MLP trained only on the measured modality, while the other dashed line (orange) shows the highest performance achieved by any model that used both measure d modalities (0.89).

Next, we considered whether it is possible to use the joint embedding methods to impute the missing modality and then classify the test cells using the MLP on the concatenated principal components of the two modalities (one measured, one imputed, Figure 2e). When only RNA is available for the test data, we used MOFA+, CGVAE, PoE, MoE and UniPort to impute the corresponding ADT profiles, and combined the measured RNA and imputed ADT profiles to be used by the cell type MLP classifier operating on the PCs of both modalities. Figure 5A shows that for MoE and PoE this approach led to higher MCC than a simple classifier trained on only RNA data. However, this approach did not outperform classification models trained on the joint space of RNA (Figure 2C) that also only require RNA measured at test time (denoted as joint unimodal in Figure 5), with the exception of CGVAE whose joint space classifier underperformed. MOFA+ did not do well in this task.

If we only use ADT data on the test set, we find that imputing RNA and then using a multi-modal model was detrimental for most models, but doing so using PoE did lead to comparable classification performance with respect to a model trained only on ADT (Figure 5B).

Because we actually have both modalities measured for all test cells, we can compare the predictions made by the multi-modal MLP when using either both measured profiles or when one of them is imputed (Table S16). When we use imputed protein profiles alongside gene expression, the multi-modal classifier predicts the same class as when using the measured protein profiles for 39.0%, 81.4%, 90.0%, 88.2% and 79.0% of the cells when the imputation is done using MOFA+, CGVAE, PoE, MoE and UniPort, respectively. The corresponding agreement rates when we combine measured protein profiles with imputed RNA profiles are 73.6%, 72.1%, 86.7% and 64.1%, respectively for MOFA+, CGVAE, PoE and MoE (Table S16). Remember that it is not possible to embed ADT values and reconstruct RNA profiles using UniPort.

We additionally used this set-up to further test how realistic the generated profiles are. If we have measured RNA at test time, we can impute the corresponding protein profiles, project them to their 32 PCs and feed them into the ADT classifier (Table S16). Although this does not have any practical utility and leads to worse performances than using the multi-modal classifier, we found that the predictions of the ADT classifier after performing this imputation matched the predictions of the same classifier made using the measured data really well for MoE and PoE, with agreements of 73.9% and 79.4%, respectively (Table S16). When doing the reverse experiment, i.e. imputing RNA from protein profiles, projecting to the RNA PCs and feeding into the RNA classifier, the agreement rates were lower, with exception of MOFA+. These agreement rates are smaller than those we found when feeding one measured and one imputed modality into the multi-modal, but they are still significantly higher than what we would expect if the classifier was randomly guessing when predicting using imputed profiles.

The relatively large correspondence between predictions made with measured and imputed profiles is additional evidence for the high imputation quality of non-linear joint embedding methods. This experiment further demonstrates the superiority of the PoE models at imputing -omics profiles (Table S16).

### Joint embedding methods scale linearly with training set size

We tested the scalability of the different methods by measuring the time required to perform one epoch of training as a function of training set size. To this end, we used random sub-sets of the CITE-Seq dataset of various sizes ranging from 5% to 100% of the full size. We observed a linear relationship between training set size and run-time for all methods (Figure S9). MoE is by far the slowest of all and has the largest slope, meaning that it consistently is slower with respect to the rest as the training set size increases. The linear method MOFA+ is surprisingly not remarkably faster than the neural network-based methods. ccVAE has the least steep slope and becomes the fastest for large datasets, but given its poor performance in imputation and classification, this is not a sufficient reason to prefer this method. PoE, which was the best model overall in terms of performance, ran at a reasonable speed, though both UniPort and totalVI were slightly faster.

## DISCUSSION

We presented a comparison of established linear joint embedding methods to novel neural-network-based methods that can learn non-linear mappings in both bulk and single-cell multi-omics data. We also included simple appropriate baselines that do not employ any joint dimensionality reduction in each experiment, which are often omitted in similar studies (e.g. [9]).

We found that non-linear methods developed in other fields (PoE and MoE) generally outperformed the linear and simple non-linear ones at imputing missing modalities. On the other hand, these methods underperformed with respect to baseline non-linear methods in terms of generation coherence. Regarding downstream supervized tasks, we observed that joint embedding can lead to improved performance when only a single modality is available in the test data, which verifies previous results for linear methods [29]. If data from both modalities are available at test time, joint embedding did not provide a significant advantage in the tasks that we tested here. Although early integration approaches were not successful at imputation, we found that totalVI outperformed ccVAE at cell type classification, which hints that there are potential benefits from the use of a conditional VAE framework. Finally, we showed that UniPort, a method originally proposed for single-cell data, can also be applied to bulk datasets, as it outperformed both MOFA+ and MCIA on all tasks on the TCGA data.

Interestingly, we additionally showed that joint embedding methods can be used to impute missing modalities which can be fed to a multi-modal classifier trained only on real data. This means that the tested models are able to generate realistic enough -omic profiles. On the other hand, these profiles were projected to the principal components space before being fed to the classifier, which provides an additional denoising step. In most cases, this approach of imputing and classifying with two modalities was worse than training a model in the joint space using one modality. This is not surprising, as imputation errors are bound to be propagated into the classifier despite the denoising. On the other hand this approach did show improvement over using a supervized model trained on a single modality.

To make sure we fairly compare all methods, we performed an extensive hyperparameter search to find the best settings for each one. We used a held-out validation set to calculate the validation loss for each hyperparameter combination and select the optimal combination. The performances of the methods were estimated using another held-out test set comprising previously unseen data points. If, for some reason, the validation loss is not predictive of the performance at a specific downstream task – as we have previously shown can be the case for VAEs trained on RNA-Seq data [30] – then the chosen hyperparameters for a given model might not be the optimal ones for the downstream task. Our results here hint that this might be the case in our setting too. Many complex models did well at imputing one modality from the other, which is part of the loss function for training them, but showed less impressive results in other downstream tasks. Therefore, if the goal of learning a joint embedding is to perform well at a specific downstream task, we recommend to employ a semi-supervized training scheme, where labeled data are used during the embedding learning process. With these labeled data one can simultaneously minimize the sum of the joint embedding loss and the supervized loss of a model trained on the target task with inputs from the joint latent space as in [31, 32].

Examining the results on generation coherence, we found that CGVAE and ccVAE did better than UniPort, PoE and MoE on both bulk and single-cell data, while they typically underperformed in the other tasks. ccVAE uses a single encoder for the concatenation of both modalities, which might be beneficial for generation coherence, as the latent space is directly and concurrently influenced by matched samples from all modalities. However, the architecture of CGVAE is identical to that of MoE and PoE with separate encoders per modality. What makes CGVAE different from these models is an additional loss term that penalizes the Wasserstein distance between the posterior distribution of the latent variables given a pair of input modalities. This encourages the latent embeddings from both modalities to be the same for the same input sample. Coherence of MoE and PoE could be potentially be improved by adding such a loss term, but that also introduces an additional hyperparameter to weigh the contribution of that loss component with respect to the total loss. Here, we used the CGVAE model as a reference, thus we did not tune this hyperparameter value.

Our results showed that our adaptation of MOFA+ [10] with out-of-sample extension was in some cases competitive with state-of-the-art neural-based embedding methods, especially in the CITE-Seq data. The addition of the linear regression on the latent variables can be seen as an additional regularization step [33] which stabilizes the model and might partly explain the good performance. In addition, MOFA+ has the advantage that it provides useful diagnostic messages about the input data as well as the learnt space. For instance, it automatically removes latent factors that explain too little variance. It would be interesting and useful to extend this concept to non-linear models and beyond the Gaussian likelihood used by MOFA+, for instance in order to detect uninformative latent variables. Here, we proposed a solution in that direction for VAE-based methods based on mutual information, which helped us classify factors as joint, modality-specific, or uninformative. Using this method, we found that joint embedding methods sometimes learn to allocate specific factors to each modality instead of finding the common biological signal.

Cantini *et al*. concluded that many of the linear joint embedding methods they tested performed well on single-cell data [9]. Here, we used a more recent single-cell dataset with many more cells and found that one of the best methods in the benchmark of Cantini *et al*., MCIA, was not even possible to train despite using a considerable amount of computational power. This points to an advantage of VAEs: they have been designed to work with stochastic or batch gradient descent to accommodate large training datasets [16]. Of the nine methods tested by Cantini *et al*. [9], only MOFA+ [10] and scikit-fusion [34] offer batch training mode and GPU acceleration and are therefore applicable to the latest generation of single-cell datasets with at least tens of thousands of cells. Many of the other methods, such as MCIA, include an eigendecomposition or singular value decomposition step, which can get very expensive for large sample and feature sizes, in terms of both time and memory. Accelerated versions of these operations (e.g. [35]) might alleviate this burden.

An important consequence of our experimental set-up is, that the number of data points used to train the joint embedding methods is equal to the number of points used to train the downstream supervised models. In practice, additional unlabeled multi-modal data might be available, which can be used during the learning of the joint space. In such cases, especially as the amount of unlabeled data increases, we expect an additional benefit of joint embedding, even if both modalities are available at test time.

Moreover, we followed the common approach of selecting the most variable features of high-dimensional modalities to include for joint embedding. In experiments on the CITE-seq dataset, we noted an association between RNA imputation performance and cell type abundance, which was partly alleviated when replacing variable genes with COSG-derived marker genes as RNA features. In addition, for some models the level-3 cell type classification also improved when using the COSG-derived marker genes, but for this classification task the best overall performances were still achieved using variable genes.

There are several possible explanations why cell type-specific marker genes show improved performance. One possible reason is that the most variable genes are indeed biased towards abundant cell types. But, likewise, another reason might be that the marker genes introduce a bias towards rare cell types, although this might be less likely because the imputation of abundant cell types also improved when using the COSG-derived marker genes. Alternatively, it might be that cell-type specific marker genes are easier to predict than the highly variable genes, because they have a more binary-like expression pattern (low in most cell types, high in one cell type or vice versa). Further exploration on the utility of exploiting prior knowledge on the cell types present in a dataset (*if available*) for feature pre-selection is a promising research direction relevant for both multi-omic and single-omic embedding methods.

As a final note, it is worth pointing out that next to learning a joint embedding space, it is interesting to learn a latent representation of the signal that is unique to each modality. In the linear setting, this has been achieved by AJIVE [12], which treats directions with significant variance that are orthogonal to the joint space as the modality-specific or ’individual’ space. When dealing with non-linear embeddings, however, finding these individual spaces is more complicated. One possible solution could be to use adversarial losses to ’force’ part of the latent space to not be useful for reconstructing the other modality.

## MATERIALS AND METHODS

### Notation

We consider datasets of $N$ samples where we measure $M$ different modalities (gene expression, DNA methylation etc.) for each sample $i$ with the $m$-th modality represented by a feature vector $\mathbf{x}^{m}_{i} \in \mathbb{R}^{D_{m}}$. We seek to find a joint latent representation for the $\mathbf{x}^{m}_{i}$’s denoted by $\mathbf{z}_{i} \in \mathbb{R}^{d}$, where $d < D_{m}$ for all $m$, which contains the common information of all modalities. The $j$-th element of $\mathbf{x}^{m}_{i}$, is denoted as $x_{ij}^{m}$. The joint embeddings are learned using a model – for example an encoder neural network – that learns the parameters $\theta _{i}$ of the distribution $p(\mathbf{z}_{i}\mid \mathbf{x}_{i}^{1},..., \mathbf{x}_{i}^{M},\theta _{i})$. For instance, the most common choice for the posterior of $z$ is a Gaussian distribution with diagonal covariance, in which case $\theta _{i}$ corresponds to $d$ mean and $d$ standard deviation values for each sample that are learned by the encoder. The entire set of samples from the $m$-th modality is represented by the matrix $\mathbf{X}^{m} \in \mathbb{R}^{N\times D_{m}}$.

In the case of autoencoder-like methods, the learned latent representations are used during training to reconstruct the input data by learning the parameters $\phi$ of the ’generative’ distribution $p(\mathbf{x}_{i}^{m}\mid \mathbf{z}_{i}, \phi _{i}^{m})$ for each modality $m$. The reconstruction quality of each modality from the latent space is assessed using the log-likelihood ($LL$) as defined in equation 1. As it is common in the literature, we assume that the likelihood factorizes over the features. The reconstructed modality is denoted as $\hat{\mathbf{X}}^{m}$, consisting of row vectors $\hat{\mathbf{x}}^{m^{T}}_{i}$. In the following, we omit the conditioning on $\theta$ and $\phi$ for simplicity. 


(1) \begin{align*}& LL(x_{i}^{m}, z_{i}) = \frac{1}{D_{m}}\sum_{j=1}^{D_{m}} log(p(x_{ij}^{m}\mid z_{i}, \phi_{i}^{m}))\end{align*}

### Algorithms for joint representation learning

We compared two linear methods, MOFA+ [10] and MCIA [11], four existing neural architectures (totalVI [17], UniPort [18], product of experts [19] and mixture of experts [20]) and two simpler, baseline non-linear joint representation learners. The six methods, visualized in Figure 1, are briefly described below. Details about training and hyperparameter optimization are provided in the Supplementary Methods.

#### Linear methods

MOFA+ infers a common low-dimensional latent space from a set of high-dimensional data modalities [10]. Each modality is mapped into the common latent space using a projection matrix $\mathbf{W}^{m}$, such that $\hat{\mathbf{x}}^{m}_{i} = \mathbf{W}^{m}\cdot \mathbf{z}_{i}$. MOFA+ makes use of prior distributions for $\mathbf{W}$ that ensure both shrinkage and differential activity of each latent dimension across modalities and employs variational inference to find the matrices that minimize the sum of the per-modality negative log-likelihoods [10]. MOFA+ only support Gaussian, Poisson and Bernoulli likelihoods.

MCIA attempts to find projections that maximize the covariance between features of each modality and a common latent space called the reference structure [11].

To allow for out-of-sample generalization for these methods, we need the projection (encoding) matrices that map a sample $\mathbf{x}_{i}^{m}$ to $\mathbf{z}_{i}$ and, as we are interested in imputing one modality from the other, we also need the inverses of these linear mappings (decoding matrices). Neither of these methods provide all the necessary matrices in their standard implementation, so we estimated them as follows: Using the training data, we fit a linear regression to predict $\mathbf{z}_{i}$ from $\mathbf{x}_{i}^{m}$, for each modality $m$ and for the concatenation of all modalities. The weights of those fitted regressions from the input data to the MOFA+/MCIA output are used as encoding matrices to embed unseen samples. We obtain the decoding matrices similarly (predicting $\mathbf{x}_{i}^{m}$ from $\mathbf{z}_{i}$), but this time we use multivariate GLMs to deal with the different distribution of each data modality (see Datasets and preprocessing).

#### Baseline non-linear embedding methods

The simplest method to combine different modalities is to concatenate the features into one vector $\mathbf{x}_{i} = [(\mathbf{x}_{i}^{1})^{T},..., (\mathbf{x}_{i}^{M})^{T}]^{T}$ and use them to train a VAE (ccVAE). The concatenated vector $\mathbf{x}_{i}$ is fed into a probabilistic encoder that learns $q(\mathbf{z}_{i}\mid \mathbf{x}_{i})$ and separate decoders are used to decode each modality from $\mathbf{z}_{i}$. The model is trained by minimizing the sum of the negative log likelihood plus a Kullback-Leibler divergence term ($KL$) between $q(\mathbf{z}_{i}\mid \mathbf{x}_{i})$ and the prior distribution of the latent variables $p(\mathbf{z})$ (equation 2). We used Gaussian distributions with diagonal covariance for both the prior and the posterior of $\mathbf{z}$ [16] 


(2) \begin{align*}& L_{ccVAE} = \sum_{i} \left[-\sum_{m=1}^{M} LL(x_{i}^{m}, z_{i}) + KL( q(z_{i}\mid x_{i}) \mid\mid p(z) ) \right]\end{align*}

Another simple non-linear joint representation learning architecture consists of one VAE for each data source. To force the encoders to embed the data in a common space we want the decoders to be able to reconstruct any modality from the latent representation of any encoder. To do so, we minimize the sum of the reconstruction losses for all possible combinations of input and output (reconstructed) modalities. We further add a loss term penalizing the second-order Wasserstein distance between the posterior distribution of $\mathbf{z}$ given each input data source. This encourages the output of different encoders (i.e. the learned embeddings of the same sample) to be similar. The loss function of this model, which we call Cross-Generating Variational Autoencoder (CGVAE), is shown in Equation 3, where $W_{2}$ is the second-order Wasserstein distance. 


(3) \begin{align*} L_{CGVAE} = \sum_{i} \left[-\sum_{m=1}^{M} \sum_{n=1}^{M} LL(x_{i}^{m}, z_{i}^{n}) +\right. \nonumber \\ \sum_{m=1}^{M} KL( q(z_{i}^{m}\mid x_{i}^{m}) \mid \mid p(z) ) + \nonumber \\ \left. \sum_{m=1}^{M-1} \sum_{n=m+1}^{M} W_{2}(q(z_{i}^{m}\mid x_{i}^{m}), q(z_{i}^{n}\mid x_{i}^{n})) \right]\end{align*}

#### Existing non-linear embedding methods

TotalVI [17] is an extension of scVI [13] for CITE-Seq data. It performs early integration by concatenating the RNA and protein data (like ccVAE) and passing them through an encoder, but uses a conditional VAE [36] to encode for covariates of interest such as library size and batch. The joint embeddings along with covariates are fed into two separate decoders to reconstruct the two modalities and the model is trained by minimizing the reconstruction loss.

UniPort was mainly designed for integrating unpaired datasets from different modalities, but it also enables joint embedding of paired data [18]. It does so by using a single encoder and two decoders. The encoder embeds one pre-selected modality (reference modality) into a latent space, which is in turn fed into two separate decoders. This forces the encoder to learn features that are predictive of both modalities, but has the downside that only the reference data can be projected into this latent space. Throughout this work, we used gene expression as the reference modality for UniPort.

The Product of Experts (PoE) approach [19] uses a single VAE per data modality, but combines all the per-modality latent representations into one final posterior distribution for $\mathbf{z}$. This distribution $q(\mathbf{z}_{i} \mid \mathbf{x}_{i}^{1},..., \mathbf{x}_{i}^{M} )$ is given by multiplying the individual densities $q(\mathbf{z}_{i}\mid \mathbf{x}_{i}^{m})$ with each other as well as with the prior $p(\mathbf{z})$. If $q$ and $p$ are chosen to be Gaussian, then the resulting product is also a Gaussian (up to a normalizing constant). In practice, PoE models are trained by sampling latent vectors from both the joint $q(\mathbf{z}_{i}\mid \mathbf{x}_{i}^{1},..., \mathbf{x}_{i}^{M} )$, as well as the individual $q(\mathbf{z}_{i}\mid \mathbf{x}_{i}^{m})$, passing each of those through all decoders and summing the different reconstruction losses. For more details on the loss function of PoE, see the original publication [19].

Mixture of Experts (MoE) [20] uses the mixture of the individual densities so that $q(\mathbf{z}_{i}\mid \mathbf{x}_{i}^{1},..., \mathbf{x}_{i}^{M} ) = \frac{1}{M}\sum _{m} {q(\mathbf{z}_{i}\mid \mathbf{x}_{i}^{m})}$. In addition, following the original publication [20], when training MoE, we replaced the Gaussian priors and posteriors of $z$ with Laplace distributions and employed the DReG gradient estimator [37] to reduce the variance of the gradient estimates.

### Datasets and pre-processing

#### TCGA dataset

The TCGA dataset [38] consists of 8440 samples of tumors – including a few adjacent normal samples as well – for which GE, CNV and DNA methylation (ME) are measured for 33 different tumor types. For GE, we used the batch-corrected data [39], standardized to zero mean and unit variance per gene with a Gaussian likelihood. For CNV, we used the per-gene copy number data [40] estimated using GISTIC2 [41]. The copy number estimates were discretized using the GISTIC2 thresholds into one of five possible categories: homozygous deletion, heterozygous deletion, normal copy number, low-level amplification and high-level amplification. As the data are discrete, we used the categorical log-likelihood. For ME [42], we restricted to samples measured with the Illumina 450K chip. We grouped CpG sites that lie within 1000 bp from the transcription start site of the 24 994 protein-coding genes (Ensembl version 79), averaging the beta values within each group ignoring missing values. If all CpG beta values of a group were missing for a particular sample, we set that feature value to 0. Finally, to accomodate the use of a beta log-likelihood, we replaced all zero’s with a small number ($\epsilon =10^{-6}$) and all one’s with $1-\epsilon$. For each data modality, we selected the 5000 most variable features based on median absolute deviation. Clinical (meta-)data for the samples were collected from [43].

#### Paired RNA and ATAC-Seq data

We downloaded a dataset of 11 910 PBMCs from https://support.10xgenomics.com/single-cell-multiome-atac-gex/datasets/1.0.0/pbmc_granulocyte_sorted_10k. We filtered out cells with fewer than 1000 or more than 25 000 measured genes and similarly cells with fewer than 5000 or more than 70 000 detected ATAC peaks. We additionally removed cells with more than 20% of their RNA-Seq reads mapping to mitochondrial genes, leaving us with 10 412 cells. We used Seurat [7] to select the 3000 most variable genes after correcting for the relationship between mean and variance using a LOESS curve and the 5000 most frequently observed ATAC peaks.

Raw RNA-Seq counts are modelled with a negative binomial log-likelihood with mean $\mu$ and dispersion $\phi$, following the modeling of totalVI [17]. The decoder predicts the value of $\mu$ for each gene in each sample, while each gene has the same dispersion parameter $\phi$ across all samples. These dispersion values are model parameters that are optimized using the Adam optimizer along with the weights of the neural network. ATAC peaks were binarized in each sample (0 reads or at least 1 read) and modelled with a Bernoulli log-likelihood.

#### CITE-Seq data

We used a CITE-Seq dataset [7] containing single-cell expression profiles for RNA and surface proteins of peripheral blood cells measured using RNA-Seq and anti-body-derived tags (ADTs) respectively. Using scvi-tools [44], we merged antibody tags targeting the same protein and removed doublets and cells with more than 12% mitochondrial-derived RNA. Additionally, we only kept cells with at least 150 detected proteins and a protein library size between 2000 and 30 000.

We selected the 5,000 most variable genes with the same approach as for the RNA+ATAC dataset above and again modelled the raw RNA counts with a negative binomial distribution with gene-specific dispersion. We retained all 217 protein features and modeled the raw counts with a two-component mixture distribution as in totalVI [17]: The first component corresponds to the non-specific binding of the ADT’s (background) and is assumed to follow a negative binomial distribution for each protein with both the mean ($\mu$) and dispersion ($\phi$) being free parameters optimized along with the network’s weights. The second component (foreground) is also a negative binomial per protein with the same $\phi$ as the background and a mean equal to $\alpha \cdot \mu$, where $\alpha$ is a number greater than 1 that is an output of the decoder for each protein and each cell. For each cell-protein pair, the decoder also outputs the mixing coefficients of the two components.

### Experiments and evaluation

#### Missing modality imputation

We split each dataset into a training, a validation and a test set. We evaluated the ability of joint embedding methods on predicting (imputing) one data modality from the other, by holding out one modality at a time from the test data (Figure 2A).

We used the log-likelihood of the held-out data given the model predictions as evaluation measure. For example, when imputing, say ME from GE, we give each GE profile ($\mathbf{x}_{i}^{GE}$) as input to the GE encoder which calculates $q(\mathbf{z}_{i}\mid \mathbf{x}_{i}^{GE})$. We take the mean of that distribution as the final embedding vector of sample $i$ and feed it to the ME decoder. The log-likelihood of the decoded ME profile then defines the performance. ccVAE requires both modalities to be fed as input, so, similar to [17], we replaced the missing modality with a vector of zeroes and passed the concatenated vector to the encoder. As a baseline imputation method, we trained a GLM to perform regression from one modality to the other using for each modality the same log-likelihood as described in the previous section.

For the TCGA data, the splits (80–10–10%) were stratified per tumor type to account for the vast imbalances between them. We trained then models using two modalities at a time: GE + ME and GE + CNV. Similarly, on the RNA+ATAC-Seq data, we performed a 80–10–10% split stratified per cell type. As the inter-individual variations are very large in the CITE-Seq dataset, to avoid information leaks stemming from using cells from the same individual during training and testing, we split the cells based on the eight donors. Specifically, we used all cells from six individuals for training, one individual for validation and one for testing. Although this split is not stratified per cell type, it provides a more realistic and less biased evaluation.

#### Generation coherence

Given a point in the latent space, we expect all decoders to generate instances of each modality with similar properties [20]. We assessed this by randomly drawing from $p(\mathbf{z})$ and checking whether the decoded profiles reflected ’similar’ samples. As these are generated profiles, we cannot check whether they come from the same individual, so we tested whether they resemble profiles from the same cancer type or cell type.

Specifically, we trained five two-layer perceptrons, one for each modality (GE, ME and CNV for TCGA, RNA and ATAC for the RNA+ATAC-Seq datasets, and RNA and ADT for CITE-Seq), where the input is a modality profile and the target output is the cancer type or the (level-2) cell type, respectively. Again using ME and GE as an example, for each model, we randomly sampled 2000 points from the prior distribution of $\mathbf{z}$ and decoded each point using both decoders to generate 2000 ME and 2000 GE matched profiles. We then fed the generated profiles into the corresponding perceptron classifier and compared the predictions of the two classifiers for the generations of the same randomly-sampled point in the latent space. Joint decoding quality, also called latent space coherence, was measured as the agreement rate of the two classifiers, i.e. the fraction of random $\mathbf{z}$’s whose decoded profiles are predicted to be from the same cancer type. This experiment is illustrated in Figure 2B.

Here, we are not interested in whether the predicted labels of the random points are correct or not, as long as the two perceptrons make the same prediction for profiles generated from the same value of $\mathbf{z}$. However, this percent agreement is only meaningful if the perceptrons are well-trained and can accurately discriminate among cancer/cell types. As we see in Table S5, the cancer type predictor trained on CNV data network was not accurate, thus we did not perform this experiment in the GE + CNV dataset.

#### Survival analysis on the TCGA dataset

We compared the latent features obtained from the different joint embedding methods on their ability to predict disease progression. To this end, we performed survival analysis using Cox regression (Figure 2C) with Progression-Free Survival (PFS) as the outcome variable [45]. We used the entire dataset to fit the models and centered and scaled each latent feature to zero mean and unit variance. To compare the generalization capability of the different models, we used the AIC [46], which is defined as $2k - 2ln(L^{s})$, where $k$ is the number of parameters the Cox model has to estimate, and $L^{s}$ is the model’s likelihood. The model with the lowest AIC value provides the best trade-off between fitting the data well and not using too many parameters. It has been shown that model selection using AIC is asymptotically equivalent to using cross-validation [47]. Therefore, the model with the lowest AIC is considered to generalize best to new, unseen data. The lower and upper bound of AIC values are specific to a dataset and a model class and impossible to calculate analytically.

To distil effects of each individual modality, this evaluation took place using the joint embeddings of either both modalities ($q(\mathbf{z}\mid \mathbf{x}_{1},\mathbf{x}_{2})$) or of only one modality at a time (e.g. $q(\mathbf{z}\mid \mathbf{x}_{1})$). See Supplementary Methods for more details.

We included the patients’ sex (2 one-hot encoded features), age, and cancer type (33 one-hot encoded binary features) as covariates in all models to account for the effects of these variables. A model with only the covariates (i.e. without any -omics features) was used as a baseline. We also compared to three stronger baselines per dataset, namely the 32 principal components of each modality and the concatenation of these pairs of 32 latent features (Figure 2D). The number of principal components was not tuned and was selected to be similar to the dimensionality of the joint embedding spaces.

#### Cell type classification on single-cell data

We followed the same experimental set-up for both the RNA+ATAC-Seq and the CITE-Seq dataset. We compared the performance of the different embeddings when both modalities are available for all samples, and when both modalities are available for the training and validation data, but only one modality is measured in the test data.

For each joint embedding method, we thus obtained three latent vectors: (1) using only RNA, (2) using only proteins (or chromatin accessibility) and 3) using both RNA and proteins (or RNA and chromatic accessibility) (Supplementary Methods).

We trained two different classifiers for each feature vector: a linear Support Vector Machine (SVM) and a two-layer perceptron. For the SVM, we chose the best value for the weight of the L2 regularization from the values [$10^{-4}$, $10^{-3}$, 0.01, 0.1, 0.5, 1.0, 2.0, 5.0, 10.0, 20.0] using the validation data and the Matthews Correlation Coefficient (MCC) as criterion. For the perceptron, we set the number of hidden neurons to 64, the learning rate to 0.0001 and trained for 150 epochs minimizing the cross-entropy loss between the ground truth and the predicted cell type labels. The epoch with the lowest validation loss was chosen for evaluating the network.

We additionally built baseline classifiers that did not use any joint embedding, but were trained on (a) the 32 principal components of the RNA data, (b) the 32 principal components of the ADT or ATAC data and (c) the concatenation of a and b. We used the same classifiers with the same settings as above and again did not tune the number of principal components for the task.

Finally, Figure 2E shows a third evaluation scheme for cell type classification. In this setting, we again assume that only one modality is measured at test time, but instead of using the joint space of that modality, we used the trained joint embedding methods to impute the missing modality for each cell. The imputed profiles are then projected to the 32 PC space and fed into the baseline multi-modal classifier (Figure 2D).

We then compare the predictions of the multi-modal classifier when using two measured modalities versus one measured and one imputed modality as well as to a classifier trained on the joint space of the measured modality (Figure 2C). We only performed this experiment on the CITE-Seq dataset.

Because we observed that predicting the level-2 assignments was a relatively easy task, in the CITE-Seq dataset (Table S5), we used the more fine-grained level-3 labeling to make the problem more challenging. The train-validation-test split was identical to the one used for the imputation experiments.

## CONCLUSION

In conclusion, both linear and non-linear joint embedding methods are applicable in many different biological problems, but it is necessary to compare them to appropriate baseline methods to identify the added effect of employing them.

**Key Points**

- Imputation of missing modalities requires non-linear modeling.
- The performance of joint embedding methods should always be compared to the performance of the concatenated principal components of each modality.
- Training a classifier in the joint space increases downstream supervized performance if only one modality is measured at test time.
- Artificial -omics profiles generated by non-linear joint embedding methods can be used for supervized tasks with limited performance drops.

## Supplementary Material

supplement_bbad416

## Data Availability

We used publically available data that can be found in the following links: TCGA: https://portal.gdc.cancer.gov/, RNA+ATAC-Seq: https://support.10xgenomics.com/single-cell-multiome-atac-gex/datasets/1.0.0/pbmc_granulocyte_sorted_10k and CITE-Seq: https://atlas.fredhutch.org/nygc/multimodal-pbmc/.

## Notes

**Stavros Makrodimitris** is a post-doctoral researcher at Erasmus University Medical Center, having obtained a doctoral degree from Delft University of Technology. His interest lies in integrating diverse data modalities and in non-invasive detection and monitoring of disease using liquid biopsies.

**Bram Pronk** is an MSc student in artificial intelligence with the bioinformatics specialization at the Delft University of Technology. His research is currently focused on functional genomics and systems biology.

**Tamim Abdelaal** obtained his PhD at the Delft Bioinformatics Lab at Delft University of Technology and later worked as a post-doc at the Department of Radiology at Leiden University Medical Center. He is currently employed as research scientist at Genmab.

**Marcel Reinders** is a professor at Delft University of Technology where he heads the Pattern Recognition and Bioinformatics group that conducts research into pattern recognition, computer vision and bioinformatics. He also has a secondary appointment with the Leiden University Medical Center, Leiden, the Netherlands, where he heads a computational biology group that translates bioinformatics towards clinical applications.
