# Supplementary material for: An in-depth comparison of linear and non-linear joint embedding methods for bulk and single-cell multi-omics
Source: Brief Bioinform. 2023 Nov 28;25(1):bbad416. doi: 10.1093/bib/bbad416 (PMC10685331; doi:10.1093/bib/bbad416)
Supplement: supplement_bbad416 [file supplement_bbad416.pdf]

An in-depth comparison of linear and non-linear joint embedding methods for bulk and single-cell multi-omics
Supplementary Material

Supplementary Methods

Hyperparameters

We trained all neural network models using PyTorch and the Adam optimizer with batch size of 64. We used the common choice of $\mathcal{N}(0, I)$ for $p(\mathbf{z})$ and a normal distribution for the variational posterior $q(\mathbf{z} \mid \mathbf{x})$ for CGVAE, ccVAE, PoE, UniPort, and totalVI. For MoE, we followed the original publication and used Laplace distributions for both the prior and the posterior. We employed a grid search for each model and in each of the three datasets to find the optimal combination of the following hyperparameters:

- learning rate ($1e-3$ or $1e-4$)
- dimensionality of z (32 or 64)
- encoder hidden layers (none, 128, 256, 256-256, 256-128)
- dropout probability (10% or no dropout)
- use of batch normalization (yes or no)

In each case the decoder architecture was symmetric to that of the encoder. MoE has one more hyperparameter: the number of samples (K) drawn from the posterior of $\mathbf{z}$. For that we tried K=10, or 20. During the grid search, we trained each configuration for a maximum of 500 epochs and applied early stopping if the validation loss did not improve for more than 0.5% for longer than 10 epochs.

totalVI and UniPort have hard-coded non-linear encoders, so that removed one option from the encoder architecture and reduced the tested hyperparameter combinations to 64. UniPort has also hard-coded dropout and batch normalization settings, further reducing the possible combinations available to 16. MOFA+ and MCIA are linear models and for those we only optimized the number of latent dimensions (32 or 64) per dataset. UniPort has also hard-coded a linear decoder which we did not change. The validation loss was the criterion to select the best combination of hyperparameters for each model in each dataset with the exception of UniPort and MOFA+, which are programmed to monitor the training loss.

Implementation details

When we pass an omic profile from modality m ($\mathbf{x}_i^m$) into the encoder network, we obtain the latent representation of that sample in the joint space of modality m via the distribution $q(\mathbf{z}_i \mid \mathbf{x}_i^m)$. Exceptions to that are ccVAE and totalVI, which use concatenated profiles from all modalities. Therefore, for those models, to calculate the parameters of $q(\mathbf{z}_i \mid \mathbf{x}_i^m)$, we set the second modality to a vector of zero's.

When both modalities are present, obtaining $q(\mathbf{z}_i \mid \mathbf{x}_i^1, \mathbf{x}_i^2)$ is straightforward for ccVAE and totalVI (profiles are concatenated and passed through the encoder). For PoE, it is obtained by multiplying the densities $q(\mathbf{z}_i \mid \mathbf{x}_i^1)$ and $q(\mathbf{z}_i \mid \mathbf{x}_i^2)$, which gives us a new Gaussian distribution. We can then obtain a single embedding for sample i by taking the mean of this distribution.

In the case of MoE, however, this is not straightforward. $q(\mathbf{z}_i \mid \mathbf{x}_i^1, \mathbf{x}_i^2)$ is a mixture of $q(\mathbf{z}_i \mid \mathbf{x}_i^1)$ and $q(\mathbf{z}_i \mid \mathbf{x}_i^2)$ and the mean of that mixture distribution might be a vector that is very improbable by both single-modality posteriors. During training, this is amended by drawing multiple samples

from the variational posterior, but for our downstream analyses we need a single feature vector per sample. CGVAE suffers from a similar issue, as its formulation does not provide a method to obtain a single joint vector based on both modalities. For these two models, we obtained a latent representation based on both modalities by concatenating the mean vectors of $q(\mathbf{z}_i \mid \mathbf{x}_i^1)$ and $q(\mathbf{z}_i \mid \mathbf{x}_i^2)$.

MCIA

We used the R package omicade4 to run MCIA. Training this joint embedding on the PBMC data was not possible due to extreme memory requirements (> 750GB), but we ran into convergence problems in the remaining datasets too. To keep the number of latent factors comparable with the neural networks, we ran MCIA with 32 and 64 latent factors, but both of these setting leads to run-time convergence errors and did not yield any output in all three datasets (GE+ME, GE+CNV, RNA+ATAC). We then started decreasing the number of factors from 32 in steps of 4 until we could obtain an output.

MOFA+

We ran MOFA+ with 32 and 64 latent factors in CPU mode (i.e. without the GPU acceleration feature), using the MOFA2 R package. All the settings were left to their default values, except for the early stopping parameter (called "convergence mode"), which we set to 'medium'. For the CITE-Seq dataset and for the timing experiments, we did use the GPU mode. MOFA+ includes a post-processing step where factors not explaining any variance are removed, which is why we got models with other than 32 or 64 factors in Tables S4, S9, and S11. We applied the built-in "select_model" function to select the best of the two models.

totalVI

Using totalVI with batch and/or individual information as covariates can enhance its performance, but this requires a fine-tuning step on the test data to allow the model to make predictions on unseen data. To ensure a fair comparison without information leaks from the test data, we assumed that all cells came from the same batch. The rest of the experiment followed the approach of ccVAE.

Numerical stability

Passing large numbers (such as RNA-Seq or ADT raw counts) through the encoders can lead to numerical issues cause divergences during training. To prevent that, we feed the log-transformed counts to the encoder, while the decoder still reconstructs the raw counts[1]. We did that for the gene expression data in RNA+ATAC-Seq dataset and for both gene and protein expression data in the CITE-Seq dataset.

Quantification of joint signal

To show whether a specific dimension (z_j) of the latent space of a model has encoded information about an input modality $\mathbf{X}^m$, we estimated their mutual information (MI) as follows:

$$
\begin{aligned}
MI(\mathbf{X}^m, z_j) &= \iint p(x^m, z_j) log(\frac{p(x^m, z_j)}{p(x^m)p(z_j)})\, dz_j\, dx^m = \\
&= \iint p(x^m)p(z_j|x^m) log(\frac{p(x^m)p(z_j|x^m)}{p(x^m)p(z_j)})\, dz_j\, dx^m = \\
&= \int p(x^m) \int p(z_j|x^m) log(\frac{p(z_j|x^m)}{p(z_j)})\, dz_j\, dx^m = \\
&= \int p(x^m) D_{KL}(p(z_j)||p(z_j|x^m))\, dx^m = \\
&= \mathbb{E}_{X^m}[D_{KL}(p(z_j)||p(z_j|x^m))]
\end{aligned}
\tag{1}
$$

We used the variational posterior $q(z_j|x^m)$ to approximate $p(z_j|x^m)$ (which makes the term inside the expectation the same as the KL regularisation term of VAEs) and furthermore used the

[1] https://docs.scvi-tools.org/en/stable/api/reference/scvi.module.VAE.html

training data to approximate the intractable expectation over the input modality. For a training set of size N this gives us:

$$MI(\mathbf{X}^m, z_j) \approx \frac{1}{N}\sum_{i=1}^{N} D_{KL}(p(z_j)||p(z_j|x_i^m)) \tag{2}$$

Directly comparing $MI(\mathbf{X}^1, z_j)$ to $MI(\mathbf{X}^2, z_j)$ is not fair, because their values depend on the entropy of $\mathbf{X}^1$ and $\mathbf{X}^2$ respectively and a modality with higher entropy can give higher mutual information. Instead, we devised a statistical test to test for each latent factor z_j whether its mutual information to each modality is statistically significant. To obtain a null distribution, we destroyed the relationship between $\mathbf{X}^m$ and z by randomly permuting the features of $\mathbf{X}^m, (m = 1, 2)$, and then feeding the perturbed data into the encoder to obtain values for the latent variables, which we used to estimate the mutual information between the randomized $\mathbf{X}^m$ and z_j. We repeated this permutation procedure 10,000 times and calculated the one-sided permutation p-values for each modality for each factor.

Cell type abundance bias in imputation of CITE-seq data

We investigated whether the association between RNA imputation performance and cell type abundance is due to bias introduced by using the 5,000 most variable features. We adopted an alternative pre-selection of genes: Using the COSG package, we identified the 30 most informative markers for each of the 30 level-2 cell types based on cosine similarity. Some genes were in the top-30 list for multiple level-2 cell types, giving us in total 819 unique marker genes.

We then re-trained the joint embeddings methods that can predict RNA from ADT (MOFA+, CGVAE, ccVAE, MoE, and PoE) using the 819 marker genes as RNA features instead of the 5,000 most variable genes. We did not optimize the models' hyperparameters again, but rather used the optimal settings from our previous search. We also re-trained the GLM baseline to predict the 819 marker genes from the ADT features. The imputation accuracy per cell type using the two feature sets is shown in Figure S7a. The Figure shows that training on the 819 marker genes does improve the median imputation performance for rare cell types, but also for the most frequent cell type (CD14+ Monocytes).

To ensure that our findings were not affected by the difference in the number of genes in the two datasets, we repeated the experiment this time comparing the 819 marker genes to the 819 most variable genes that were not labeled as markers (Figure S7b). The models were not retrained on 819 most variable genes not labeled as markers, instead their performance was taken from the model that included all 5,000 genes. The results are very similar when compared to those in Figure S7a, further solidifying our findings.

Time benchmarking

We compared the time it takes to run one training epoch for MOFA+, CGVAE, ccVAE, PoE, MoE, UniPort and totalVI as a function of training set size using the CITE-Seq data. We excluded MCIA from this because it does not do epoch-based training and does not run in our system for this large dataset. Next to the original dataset with 117,730 cells in the training set, we also trained on sub-sampled versions with 5%, 10%, 20% and 50% of the training data. We trained all methods using their optimal hyperparameter settings (Table S11) for 10 epochs on the same system with 2 CPUs and 1 NVIDIA Tesla P100 GPU. We ignored the first epoch as GPUs often require a few "warm-up" iterations before achieving steady state performance and recorded the run time of epochs 2-10. For the neural network models we used python3.11 and pytorch 2.0, while for MOFA+ we used the python virtual environment recommended by the developer of the package. Figure S9 shows the runtime of each model as a function of training set size.

We quantified the run-time of one iteration on the training dataset (epoch) for each tool, but there are many factors that affect the total time it takes the user to run each tool that are hard to take into account in our benchmark: First, the total number of iterations until convergence depends on many variables, such as learning rate, learning rate schedule, and batch size. Also, for a larger dataset the parameters are updated more times per epoch (because there are more batches) and that means that the number of epochs required for convergence might be smaller for larger datasets. Second, the optimal architecture for each method might be different and what is the optimal architecture can highly depend on training set size (e.g. depth of encoder/decoder).

Third, an important factor is whether the training data fit in memory. Pre-loading all data into memory accelerates the training time remarkably compared to loading each batch from the disk each time, but this of course comes at the cost of O(N) memory. In our system, it was possible to pre-load everything into memory, but this will not be possible for a dataset with e.g. 1 million cells. Then disk speed starts becoming an influential factor in training time. Despite these limitations, our benchmarking still provides valuable information on the scalability of the tested methods.

Supplementary Figures

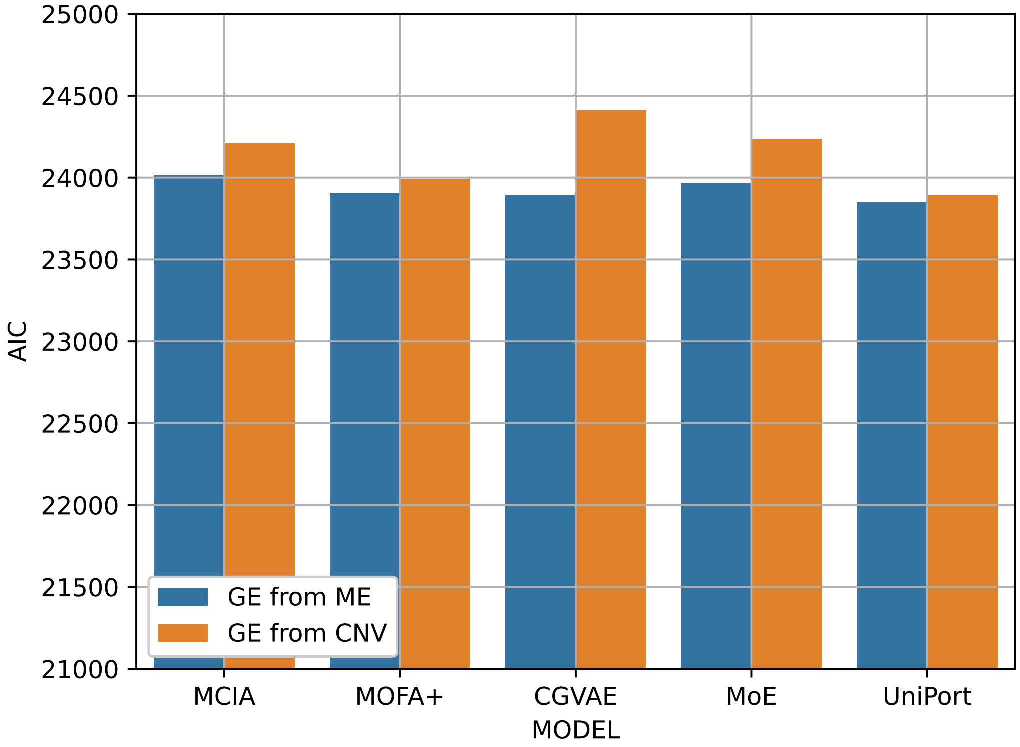

Figure S1: Predictive performance (AIC, $y-$axis) of progression-free survival of gene expression data trained in the joint space of gene expression and methylation (blue) or gene expression and copy number(orange) based on different joint embedding methods ($x-$axis).

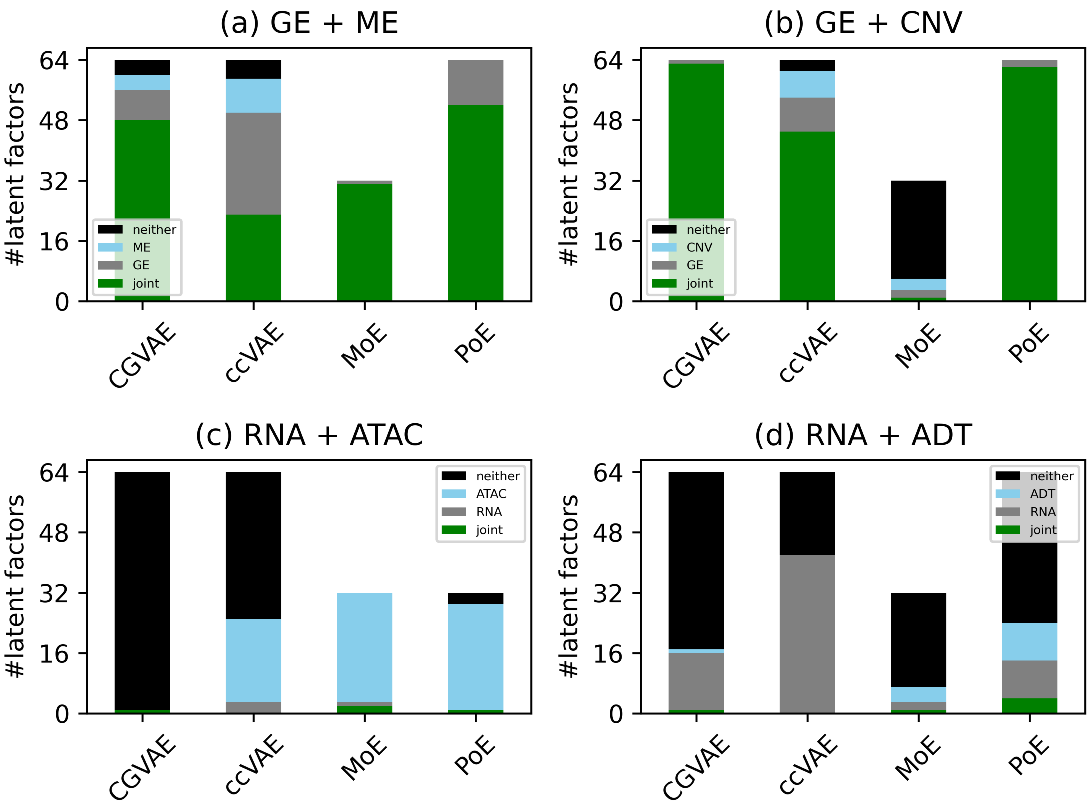

Figure S2: For each model (x-axis) we show the number of latent factors (number of neurons in bottleneck layer, y-axis), that have significantly high MI with both input modalities (joint, green), only one modality (gray and cyan), and neither modality (black) for the following datasets: (a) TCGA GE + ME, (b) TCGA GE + CNV, (c) RNA + ATAC-Seq, (d) CITE-Seq.

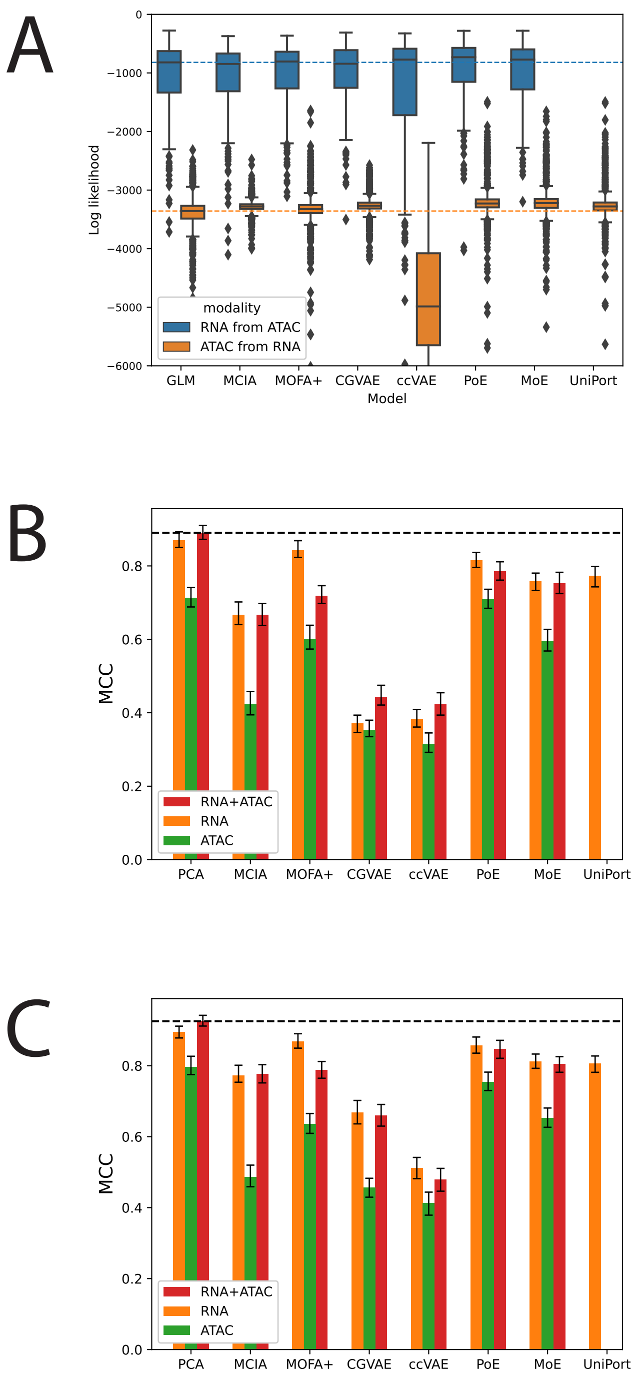

Figure S3: Evaluation on paired single-cell RNA-Seq and ATAC-Seq data. (A) Missing modality imputation performance for gene expression (RNA) from chromatin accessiblity (ATAC) and vice-versa. Performance is measured as the log-likelihood ($y-$axis) of the test samples (cells) given the predictions of each model ($x-$axis) for those data (higher is better). The distribution of the per-cell log-likelihoods is shown. The dashed horizontal lines represent the performance of the baseline GLM. Cells further than 1.5 times the interquartile range from the median are marked as outliers. (B) Cell type classification performance (MCC, $y-$axis, higher is better) achieved by training a support vector machine (SVM) in the joint space of the different models when using: only gene expression (RNA, orange), only chromatin accessibility (ATAC, green), and both RNA and ATAC data (red). The error bars denote 95% confidence intervals calculated by bootstrapping the test cells 100 times. (C) As in (B), but for a multilayer perceptron (MLP) classifier.

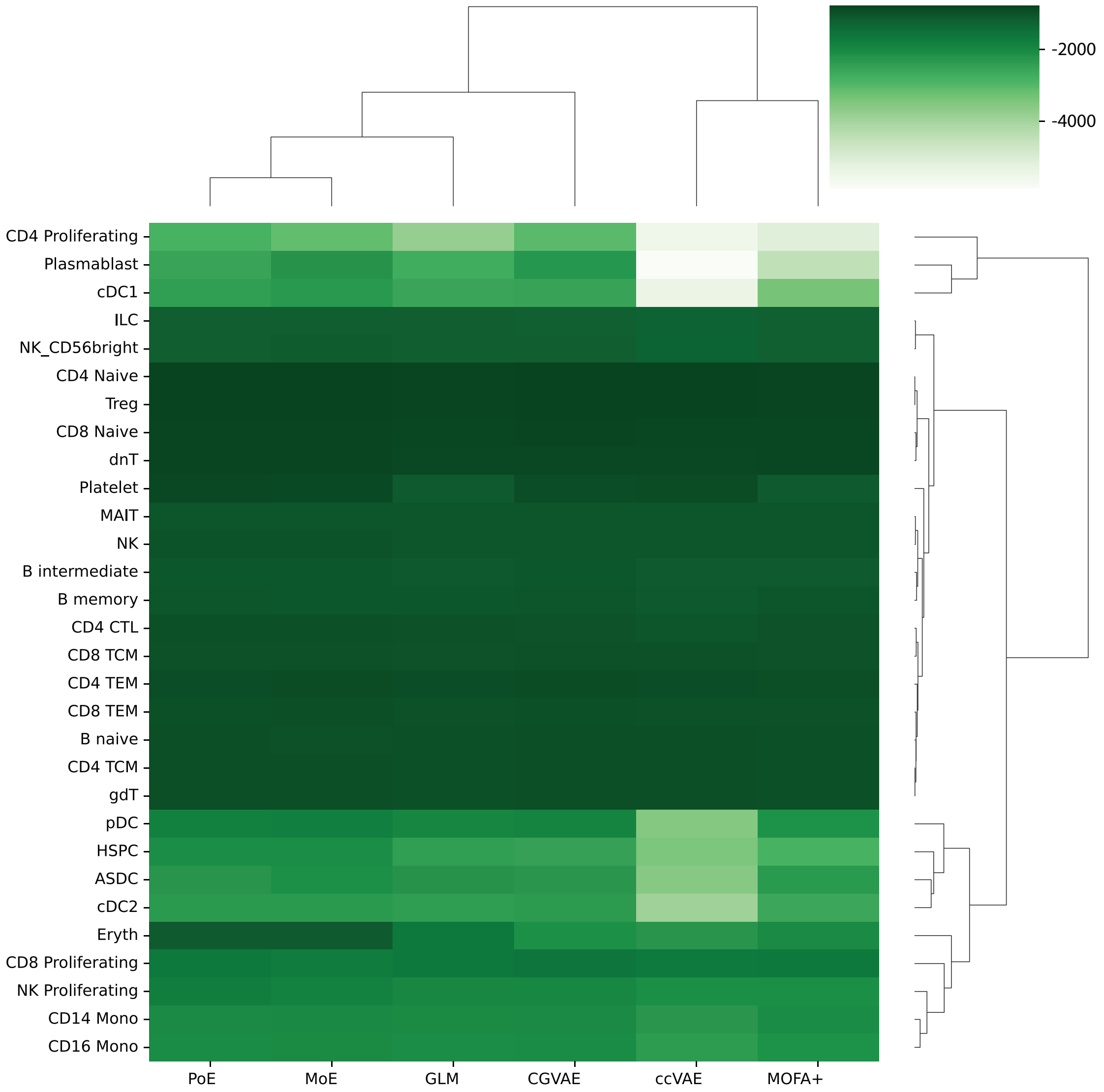

Figure S4: Log likelihood of imputing RNA from ADT for each model. The average log-likelihood is calculated per cell type for each model. Models and cell types are clustered so that similar models/cell types are next to each other. Higher log-likelihood (deeper green) corresponds to better performance.

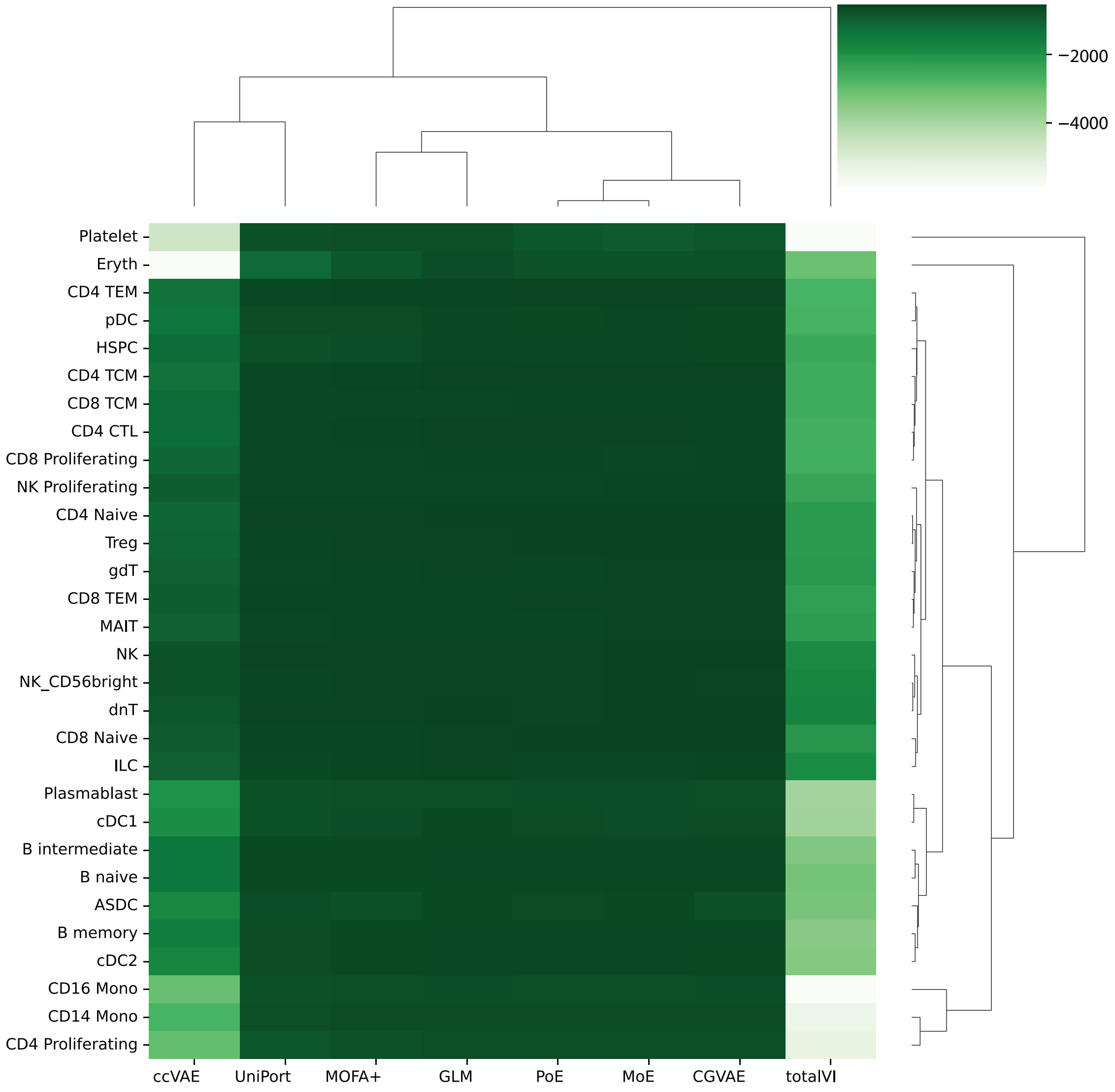

Figure S5: Log likelihood of imputing ADT from RNA for each model. The average log-likelihood is calculated per cell type for each model. Models and cell types are clustered so that similar models/cell types are next to each other. Higher log-likelihood (deeper green) corresponds to better performance.

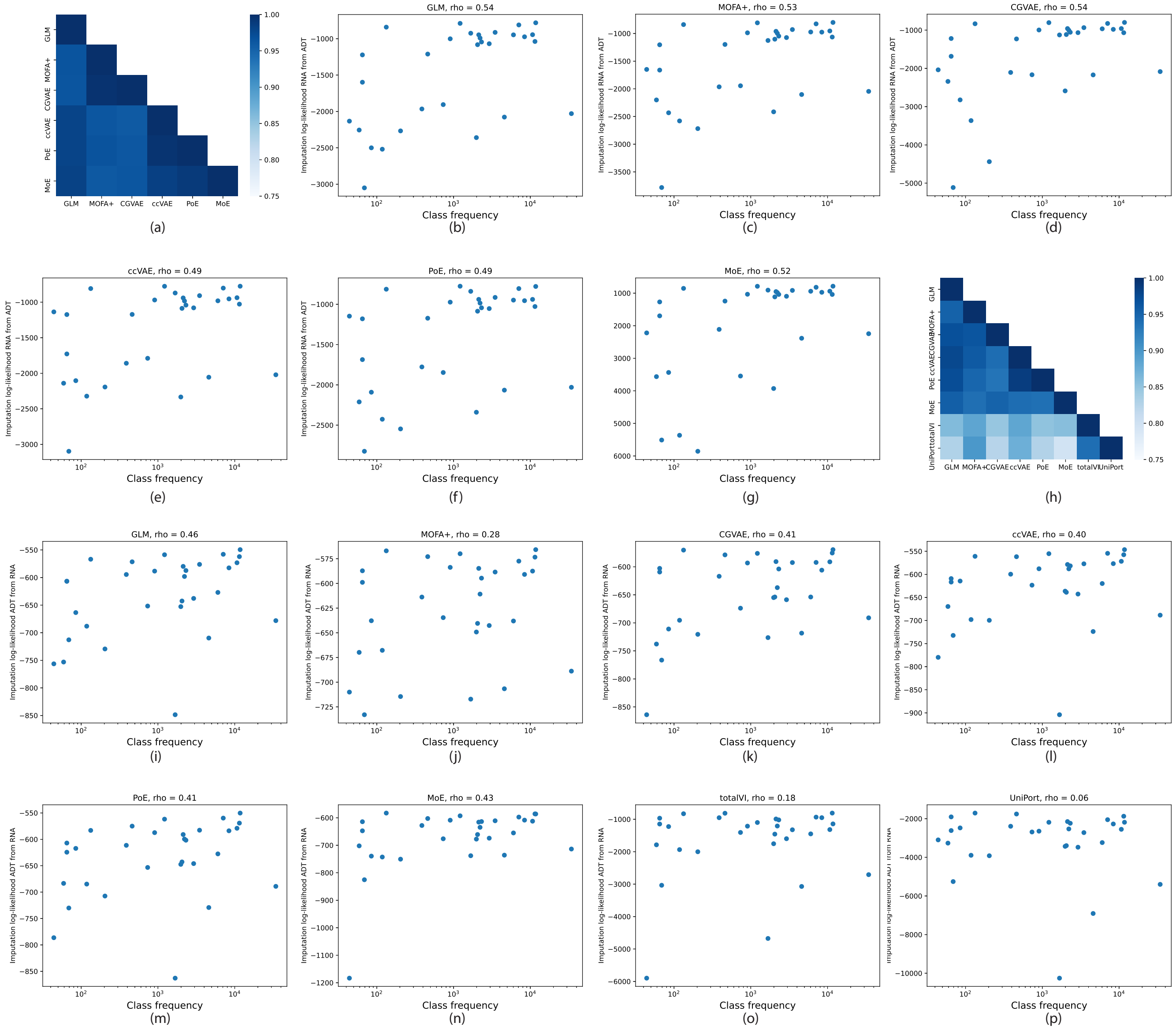

Figure S6: (a) Spearman correlation of the median imputation log-likelihood of the different methods across 30 cell types when imputing RNA from ADT. Darker blue indicates higher correlation. (b-g) Effect of cell type abundance (x-axis) on the median imputation log-likelihood (y-axis) of RNA from ADT for the GLM (b), MOFA+ (c), CGVAE (d), ccVAE (e), PoE (f), and MoE (g). (h) Spearman correlation of the median imputation log-likelihood of the different methods across 30 cell types when imputing ADT from RNA. Darker blue indicates higher correlation. (i-p) Effect of cell type abundance (x-axis) on the median imputation log-likelihood (y-axis) of ADT from RNA for the GLM (i), MOFA+ (j), CGVAE (k), ccVAE (l), PoE (m), MoE (n), totalVI (o), and UniPort (p).

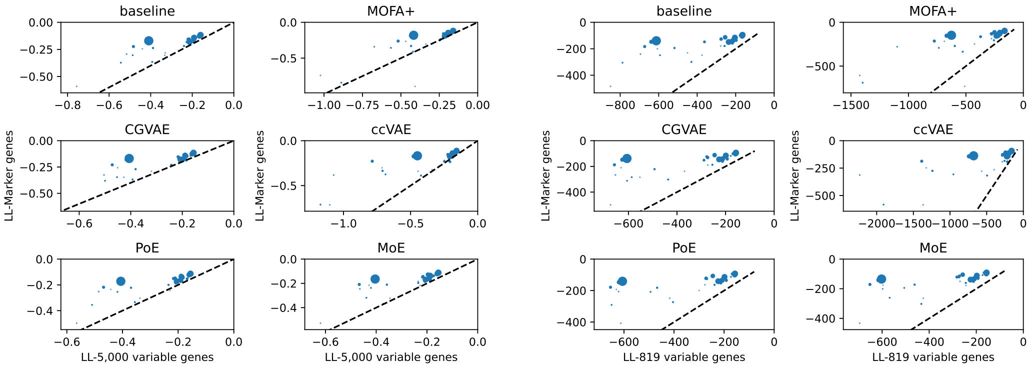

(a) (b)

Figure S7: (a) Imputation of RNA from ADT on the test set of the CITE-Seq dataset by 6 models trained using 5,000 most variable genes (x-axis) of 819 COSG-derived marker genes (y-axis) as RNA features. Performance is measured as the mean log-likelihood of a test cell across all genes. Cells of the same cell type are then aggregated using their median value to reach one average performance for each cell type (dot). The cell type abundance in the dataset is signified by the size of the dot. (b) As in (a), but the x-axis shows the performance on the 819 most variable genes that were not identified as markers by COSG.

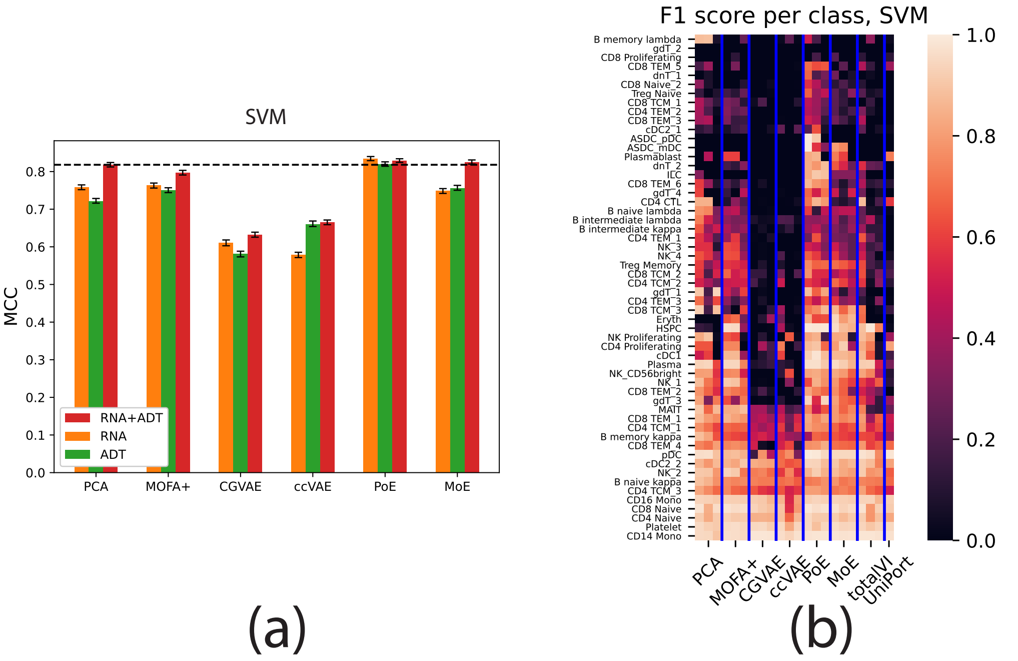

Figure S8: Cell type classification performance on the CITE-Seq dataset, when using a linear SVM classifier instead of a MLP. (a) Classification performance (MCC, $y-$axis, higher is better) achieved by training a linear SVM in the joint space of the different models when using: only gene expression (RNA, orange), only protein expression (ADT, green), and both RNA and ADT data (red). The error bars denote 95% confidence intervals calculated by bootstrapping the test cells 100 times. (b) Per-class (cell type) performance of the same classifiers. Brighter colors denote a higher per-class F1 score and therefore better performance. For each model we show three columns (RNA+ADT, RNA only and ADT only). Note that class CD4+ Tem_4 is not present in the test data and therefore not shown in the per-class evaluations (because its precision and recall is always 0 and the F1 score is thus undefined), but it was taken into account when calculating the MCC in (a).

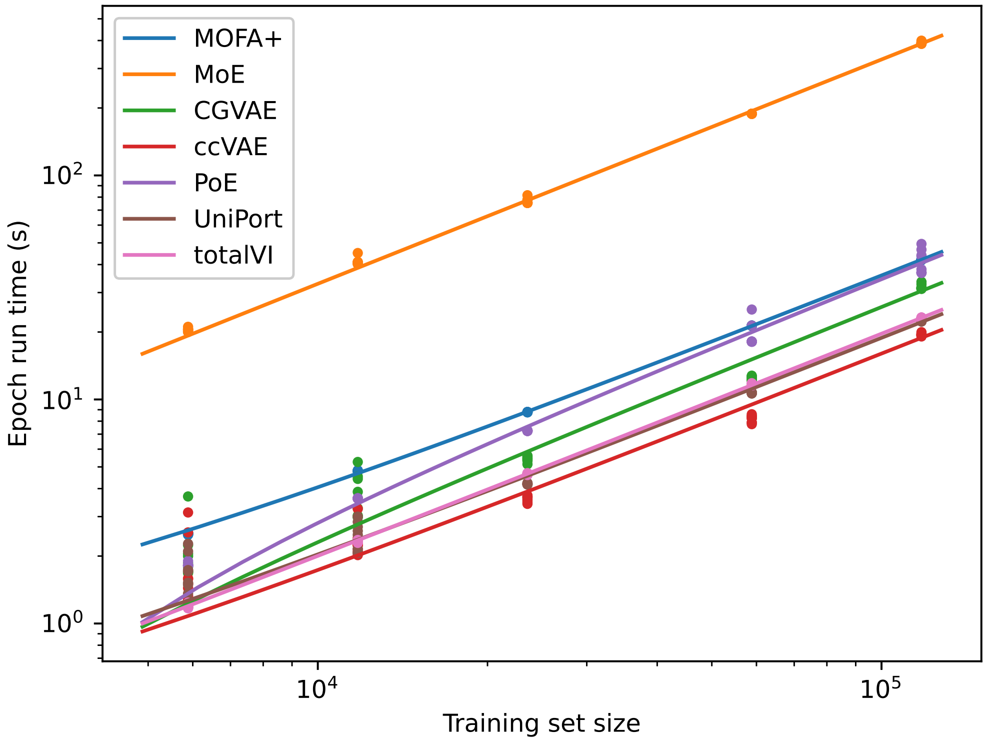

Figure S9: Run-time comparison. Each dot corresponds to one epoch of training with the $x-$coordinate corresponding to the size of the dataset and the $y-$coordinate to the run-time in seconds. Both axes are in logarithimic scale and the different dot colors designate the different models. For each joint embedding method we recorded run-time for 9 epochs per training set size and used all the points to fit a linear regression. The fitted regression line for each method shown with the corresponding color.

Supplementary Tables

Table S1: Basic features (columns) of the joint embedding methods (rows) included in this comparison. Note that in UniPort both modalities need to have the same likelihood, while totalVI uses negative binomial likelihood (N.B.) for RNA and negative binomial mixture likelihood for ADT.

model	pre-processing	likelihoods supported	loss function	joint embedding method	built-in imputation	batch gradient descent	GPU
MCIA	0-1 scaling	-	covariance	maximal covariance	No	No	No
MOFA+	-	Gaussian, Poisson, Bernoulli	log-likelihood	concatenation	No	Yes	Yes
CGVAE	-	any	log-likelihood	imputation +Wasserstein	Yes	Yes	Yes
ccVAE	-	any	log-likelihood	concatenation	No	Yes	Yes
PoE	-	any	log-likelihood	product of experts	Yes	Yes	Yes
MoE	-	any	log-likelihood	mixture of experts	Yes	Yes	Yes
UniPort	0-1 scaling	Gaussian, Laplace, Bernoulli	reconstruction	imputation	for non-reference modality	Yes	Yes
totalVI	-	N.B., N.B. mixture	log-likelihood	concatenation	No	Yes	Yes

Table S2: Mean imputation log-likelihood of gene expression (GE) from DNA methylation (ME) and vice-versa on the TCGA dataset. Higher log-likelihood is better irrespective of the sign. The values for both the validation and the test data are listed.

	VALIDATION SET		TEST SET	
model	GE from ME	ME from GE	GE from ME	ME from GE
GLM	-4,193.94	1,950.64	-4,201.91	1,805.08
MCIA	-5,000.33	4,323.83	-4,980.13	4,335.12
MOFA+	-5,376.41	4,340.35	-5,368.54	4,306.57
CGVAE	-4,162.21	5,119.92	-4,149.46	5,062.66
ccVAE	-6,573.50	2,183.08	-6,682.89	2,220.33
PoE	-3,763.50	5,517.53	-3,787.29	5,493.66
MoE	-3,861.87	5,183.17	-3,787.29	5,183.18
UniPort	N/A	5,028.98	N/A	4,996.28

Table S3: Mean imputation log-likelihood of gene expression (GE) from copy number (CNV) and vice-versa on the TCGA dataset. Higher log-likelihood is better irrespective of the sign. The values for both the validation and the test data are listed. A star (*) next to the MOFA+ values designates numerical overflow during the calculation of the mean caused by negative numbers with very large absolute values.

	VALIDATION SET		TEST SET	
model	GE from CNV	CNV from GE	GE from CNV	CNV from GE
GLM	-7,163.24	-3,957.34	-7,151.23	-4,045.75
MCIA	-11,571.34	-4,955.23	-9,880.65	-4,961.50
MOFA+	*	-6,436.83	*	-6,641.54
CGVAE	-6,954.06	-3,719.06	-6,944.19	-3,813.09
ccVAE	-28,447.95	-11,544.68	-30,851.33	-11,441.40
PoE	-6,599.93	-3,661.11	-6,555.80	-3,736.60
MoE	-7,112.39	-8,050.70	-7,116.67	-8050.665
UniPort	N/A	-3,464.99	N/A	-3,500.61

Table S4: Optimal hyperparamaters for TCGA dataset based on validation loss

dataset	model	latent dimension	encoder layers	learning rate	dropout	batch normalization	K
GE + ME	MCIA	20	-	-	-	-	-
	MOFA+	47	-	-	-	-	-
	CGVAE	64	256-256	0.001	0%	Yes	-
	ccVAE	64	256-256	0.001	0%	Yes	-
	PoE	64	256	0.0001	10%	No	-
	MoE	32	256-256	0.0001	0%	No	10
	UniPort	64	256	0.001	-	-	-
GE + CNV	MCIA	16	-	-	-	-	-
	MOFA+	60	-	-	-	-	-
	CGVAE	64	256-128	0.0001	0%	No	-
	ccVAE	64	256	0.001	0%	Yes	-
	PoE	64	256	0.001	10%	Yes	-
	MoE	32	256-128	0.0001	10%	No	20
	UniPort	64	256	0.0001	-	-	-

Table S5: Validation and test performance (Matthews Correlation Coefficient - MCC) of neural networks that predict tumor type from an input omic profile on the TCGA, (level-1) cell type from an input single-cell profile on the RNA+ATAC-Seq data, and (level-2) cell type from an input single-cell profile on the CITE-Seq data. MCC of 0 corresponds to random guessing and MCC of 1 to perfect classification.

Dataset	Data type	validation MCC	test MCC
TCGA	GE	0.968	0.958
TCGA	ME	0.962	0.955
TCGA	CNV	0.406	0.440
CITE-Seq	RNA	0.929	0.915
CITE-Seq	ADT	0.936	0.904
RNA+ATAC	RNA	0.955	0.964
RNA+ATAC	ATAC	0.875	0.868

Table S6: Survival analysis performance of different joint embedding methods (rows) on the GE+ME dataset. Lower Akaike Information Criterion (AIC) values designate better performance.

Model	Modality	AIC
covariates only	-	24,316.76
PCA	GE	23,936.77
	ME	24,034.69
	GE+ME	23,858.65
MCIA	GE	24,015.08
	ME	24,024.65
	GE+ME	24,020.04
MOFA+	GE	23,904.53
	ME	23,937.95
	GE+ME	23,918.48
CGVAE	GE	23,890.94
	ME	23,883.19
	GE+ME	23,870.96
ccVAE	GE+ME	23,860.87
PoE	GE+ME	23,906.31
MoE	GE	23,966.63
	ME	23,938.41
	GE+ME	23,911.54
UniPort	GE	23,848.98

Table S7: Survival analysis performance of different joint embedding methods (rows) on the GE+CNV dataset. Lower Akaike Information Criterion (AIC) values designate better performance.

Model	Modality	AIC
covariates only	-	24,316.76
PCA	GE	23,936.77
	CNV	24,283.20
	GE+CNV	23,947.96
MCIA	GE	24,211.88
	CNV	24,355.63
	GE+CNV	24,306.60
MOFA+	GE	23,992.16
	CNV	24,443.58
	GE+CNV	24,297.19
CGVAE	GE	24,414.22
	CNV	24,253.06
	GE+CNV	24,525.55
ccVAE	GE+CNV	23,991.02
PoE	GE+CNV	23,920.78
MoE	GE	24,237.80
	CNV	24,262.78
	GE+CNV	24,204.19
UniPort	GE	23,892.21

Table S8: Mean imputation log-likelihood of gene expression (RNA) from chromatin accessibility (ATAC) and vice-versa. Higher log-likelihood is better irrespective of the sign. The values for both the validation and the test data are listed.

	VALIDATION SET		TEST SET	
model	RNA from ATAC	ATAC from RNA	RNA from ATAC	ATAC from RNA
GLM	-1,002.85	-3,467.8	-1,004.71	-3,444.44
MCIA	-1,016.02	-3,292.57	-1,012.27	-3,313.73
MOFA+	-966.82	-3,327.05	-968.01	-3,383.72
CGVAE	-958.18	-3,270.56	-955.71	-3,263.89
ccVAE	-1,189.32	-4,911.53	-1,195.06	-4,886.56
PoE	-880.08	-3,207.82	-878.89	-3,207.64
MoE	-948.32	-3,206.31	-945.86	-3,211.83
UniPort	N/A	-3,262.18	N/A	-3,259.65

Table S9: Optimal hyperparamaters for the RNA+ATAC-Seq dataset based on validation loss

model	latent dimension	encoder layers	learning rate	dropout	batch normalization	K
MCIA	20	-	-	-	-	-
MOFA+	31	-	-	-	-	-
CGVAE	64	256-128	0.0001	0%	No	-
ccVAE	64	256-256	0.001	0%	No	-
PoE	32	256-128	0.001	10%	Yes	-
MoE	32	256-256	0.0001	0%	No	20
UniPort	32	256-128	0.0001	-	-	-

Table S10: Cell type classification performance (Matthews Correlation Coefficient) on the RNA+ATAC-Seq dataset

Model	Modality	SVM MCC	MLP MCC
PCA	RNA	0.869	0.895
	ATAC	0.713	0.796
	RNA+ATAC	0.890	0.925
MOFA+	RNA	0.843	0.868
	ATAC	0.601	0.635
	RNA+ATAC	0.719	0.788
MCIA	RNA	0.667	0.773
	ATAC	0.423	0.487
	RNA+ATAC	0.667	0.776
CGVAE	RNA	0.371	0.668
	ATAC	0.353	0.457
	RNA+ATAC	0.444	0.660
ccVAE	RNA	0.383	0.511
	ATAC	0.316	0.412
	RNA+ATAC	0.423	0.479
PoE	RNA	0.816	0.857
	ATAC	0.709	0.754
	RNA+ATAC	0.786	0.846
MoE	RNA	0.758	0.812
	ATAC	0.595	0.652
	RNA+ATAC	0.753	0.805
UniPort	RNA	0.773	0.807

Table S11: Optimal hyperparamaters for CITE-Seq dataset based on validation loss

model	latent dimension	encoder layers	learning rate	dropout	batch normalization	K
MCIA	-	-	-	-	-	-
MOFA+	49	-	-	-	-	-
CGVAE	64	256-128	0.0001	0%	Yes	-
ccVAE	64	256-256	0.0001	0%	Yes	-
PoE	64	256	0.001	0%	No	-
MoE	32	256-256	0.0001	0%	No	10
UniPort	32	256-128	0.0001	-	-	-
totalVI	32	256-256	0.001	10%	Yes	-

Table S12: Mean imputation log-likelihood of gene expression (RNA) from protein expression (ADT) and vice-versa on the CITE-Seq dataset. Higher log-likelihood is better irrespective of the sign. The values for both the validation and the test data are listed.

	VALIDATION SET		TEST SET	
model	RNA from ADT	ADT from RNA	RNA from ADT	ADT from RNA
GLM	-1216.36	-633.75	-1317.09	-633.84
MOFA+	-1243.23	-647.3	-1352.07	-645.58
CGVAE	-1194.25	-629.42	-1299.61	-631.68
ccVAE	-1311.84	-1639.8	-1437.10	-1740.90
PoE	-1181.92	-645.98	-1355.29	-682.58
MoE	-1182.54	-638.41	-1282.69	-641.06
totalVI	N/A	-3398.36	N/A	-3580.67
UniPort	N/A	-659.50	N/A	-661.06

Table S13: Contingency table showing the enrichment of marker genes in the set of 5,000 most variable genes on the RNA data of the CITE-Seq dataset.

	marker	not marker	total
most variable	694	4306	5000
not most variable	125	16423	16548
total	819	20729	21548

Table S14: Cell type classification performance (Matthews Correlation Coefficient, higher is better) on the CITE-Seq dataset

Model	Modality	SVM MCC	MLP MCC
PCA	RNA	0.756	0.807
	ADT	0.719	0.809
	RNA+ADT	0.829	0.891
MOFA+	RNA	0.775	0.858
	ADT	0.742	0.833
	RNA+ADT	0.788	0.875
CGVAE	RNA	0.610	0.732
	ADT	0.600	0.734
	RNA+ADT	0.632	0.747
ccVAE	RNA	0.527	0.785
	ADT	0.591	0.739
	RNA+ADT	0.645	0.771
PoE	RNA	0.827	0.867
	ADT	0.821	0.831
	RNA+ADT	0.840	0.883
MoE	RNA	0.742	0.828
	ADT	0.731	0.811
	RNA+ADT	0.788	0.865
totalVI	RNA	0.669	0.823
	ADT	0.684	0.755
	RNA+ADT	0.797	0.854
UniPort	RNA	0.638	0.726

Table S15: Cell type classification performance (MCC, higher is better) when using the 5,000 most variable genes and the 819 cell type marker genes as RNA features.

Level-3 labels	Modality	MCC 819 Markers	MCC 5,000 Most Variable
CGVAE	RNA	0.744	0.732
	ADT	0.750	0.734
	RNA+ADT	0.810	0.747
ccVAE	RNA	0.782	0.785
	ADT	0.751	0.739
	RNA+ADT	0.796	0.771
MoE	RNA	0.840	0.828
	ADT	0.829	0.811
	RNA+ADT	0.879	0.865
PoE	RNA	0.845	0.867
	ADT	0.841	0.831
	RNA+ADT	0.889	0.883
PCA	RNA	0.805	0.807
	ADT	0.814	0.809
	RNA+ADT	0.880	0.891

Table S16: Percent agreement of the MLP predictions using a measured profile and an imputed profile using a single-modal or multi-modal classifier trained solely on real data.

	MOFA+	CGVAE	PoE	MoE	UniPort
RNA from ADT (unimodal)	57.3%	58.0%	75.0%	35.1%	N/A
ADT from RNA (unimodal)	39.3%	61.9%	79.4%	73.9%	32.9%
RNA from ADT (multimodal)	73.6%	72.1%	86.7%	64.1%	N/A
ADT from RNA (multimodal)	39.0%	81.4%	90.0%	88.2%	79.0%
